# Changes in vertical skeletal and neuromuscular balance in growing patients treated with AMCOP®: a cephalometric and EMG evaluation

**DOI:** 10.3389/fdmed.2025.1741153

**Published:** 2026-01-06

**Authors:** Gianna Dipalma, Grazia Marinelli, Angela Di Noia, Laura Ferrante, Filippo Cardarelli, Francesco Inchingolo, Andrea Palermo, Daniela Di Venere, Angelo Michele Inchingolo, Alessio Danilo Inchingolo

**Affiliations:** 1 Interdisciplinary Department of Medicine, University of Bari “Aldo Moro”, Bari, Italy; 2 Department of Biomedical, Surgical and Dental Sciences, Milan University, Milan, Italy; 3 Department of Experimental Medicine, University of Salento, Lecce, Italy

**Keywords:** AMCOP®, elastodontic appliances, cephalometric values, Deltadent®, sEMG

## Abstract

**Aim:**

To evaluate changes in vertical skeletal dimensions and neuromuscular balance in growing patients treated with AMCOP® elastodontic appliances, by comparing pre- and post-treatment cephalometric values (Deltadent®) and standardized surface EMG indices.

**Materials and methods:**

This monocentric retrospective case series included 9 consecutive children in deciduous/early mixed dentition treated with AMCOP® according to a staged protocol (Open phase for vertical control, then class-specific device when indicated). Wear was prescribed 1 h/day plus nocturnal use. Lateral cephalograms were traced in Deltadent® at baseline (T0) and after therapy (T1). Primary outcomes were overbite and vertical divergence (SN-GoGn; PP-MP). Secondary outcomes included ANS-Me, overjet, interincisal angle, U1-PP and L1-MP. Neuromuscular balance was assessed with Teethan® (POC TA/MM, BAR, TORS, IMP, ASIM), recorded per SENIAM recommendations. Reliability was checked with ICC and Dahlberg's error; paired comparisons used standard parametric/non-parametric tests (*α* = 0.05).

**Results:**

Treatment was completed without adverse events (median duration ≈12–16 months). Most patients showed closure or reduction of anterior open bite, decreased or well-controlled vertical divergence, increased interincisal angle, and reduced overjet, with upper incisor uprighting and stable lower incisor inclination. sEMG demonstrated consistent improvement: barycenter (BAR) shifted toward the normative zone, torsion/asymmetry indices decreased, and global efficiency (IMP) increased.

**Conclusions:**

In growing patients, AMCOP® therapy was associated with favorable vertical control and measurable neuromuscular rebalancing, documented by objective cephalometric and EMG metrics. Prospective controlled studies are warranted to confirm efficacy and long-term stability.

## 1 Introduction

### 1.1 Vertical skeletal discrepancies and the challenge of anterior open bite

Vertical skeletal imbalances, particularly anterior open bites, were investigated in the current study. Anterior open bite is defined as the lack of vertical overlap between the maxillary and mandibular incisors during occlusion and represents a complex diagnostic and therapeutic challenge in pediatric orthodontics (1–13). It may result from altered skeletal growth, dental malposition, or functional factors, most notably chronic oral habits such as thumb sucking, tongue thrust, mouth breathing, and neuromuscular dysfunction (14–19). These influences, if present during critical phases of craniofacial development, can contribute to increased lower facial height and clockwise mandibular rotation, compromising facial harmony and incisal function. In addition to aesthetic consequences, anterior open bite may impair speech and lead to functional instability (20–27). Conventional orthodontic approaches often involve the use of high-pull headgear, vertical elastics, multibracket systems, or habit-breaking devices like tongue cribs. In more severe cases, surgical intervention may be considered (6, 28–34). However, these techniques often focus on the symptomatic correction of malocclusion rather than the underlying neuromuscular imbalance, increasing the risk of relapse. The AMCOP® system provides an integrative alternative that incorporates vertical control elements such as anterior bite blocks, occlusal platforms, and tongue positioners (35–43). These modules allow individualized correction of vertical discrepancies and promote neuromuscular adaptation, particularly when introduced during early mixed dentition. In the current study, eight patients exhibiting anterior open bite or vertical overdevelopment underwent treatment with AMCOP® appliances (44–51). More recently, functional orthopedic solutions such as the AMCOP® system (Modular Orthopaedic Restraint Appliances Customised), have gained attention for their capacity to combine skeletal modulation with neuromuscular rehabilitation. The AMCOP® device is removable, modular, and customizable (52–59). It stimulates natural growth by restoring proper function in the orofacial system, targeting tongue posture, mandibular position, and breathing patterns (60). Vertical skeletal evaluation was conducted using cephalometric lateral radiographs, analyzed with the digital tracing tools of Deltadent®. Parameters assessed included the SN-GoGn angle, ANS-Me distance, and overbite depth. Improvements in vertical dimensions and incisor contact were observed pre and post-treatment in patients showing favorable neuromuscular adaptation (61–66).

### 1.2 Diagnostic technologies and rationale for the present study

Despite the growing clinical use of elastodontic appliances, objective evidence evaluating their effects on both skeletal and neuromuscular parameters in growing patients remains limited. Most published studies focus primarily on dental or skeletal outcomes assessed by conventional cephalometry, while few investigations have explored the neuromuscular component using surface electromyography (sEMG), particularly in pediatric populations (67–74). Moreover, even fewer studies have adopted an integrated diagnostic approach combining digital cephalometric analysis with standardized neuromuscular assessment (75–85). Therefore, a clear gap exists in the literature concerning the simultaneous evaluation of skeletal adaptations and functional neuromuscular rebalancing induced by elastodontic therapy during growth Despite the increasing clinical use of elastodontic appliances, most available studies focus exclusively on skeletal or dental outcomes evaluated through conventional cephalometric analysis. Conversely, investigations specifically addressing neuromuscular adaptations assessed by surface electromyography (sEMG) remain limited and are often performed in adult populations or within isolated functional protocols rather than comprehensive orthodontic treatment approaches. To date, few studies have integrated digital cephalometric evaluation with standardized sEMG analysis in growing patients undergoing elastodontic therapy, and no consistent evidence is available describing the simultaneous relationship between skeletal modifications and neuromuscular rebalancing during developmental stages (86–97). Therefore, an important gap persists in the literature regarding the combined objective assessment of morphological and functional responses to elastodontic treatment in pediatric patients. The novelty of the present study lies in the integrated use of digital cephalometry (Deltadent®) and standardized surface electromyography (Teethan®) to simultaneously document vertical skeletal changes and neuromuscular adaptations in growing subjects treated with AMCOP® (98–108). This combined diagnostic approach allows the correlation of structural modifications with functional muscle activity, providing new evidence on how elastodontic therapy may influence both craniofacial growth and neuromuscular balance beyond purely dentoalveolar effects. The aim of the present study is to address this gap by assessing the effects of AMCOP® treatment on vertical skeletal parameters using digital cephalometry (Deltadent®) and on neuromuscular balance using standardized sEMG recordings (Teethan®). By integrating morphologic and functional analyses, this work seeks to provide objective data on the effectiveness of elastodontic therapy as an early interceptive strategy aimed at promoting both structural correction and functional reeducation in growing patients.

## 2 Materials and methods

### 2.1 Study design and ethical approval

This was a retrospective single-arm case series of growing patients treated for anterior open bite with elastodontic appliances (AMCOP® “OPEN”). Lateral cephalograms taken before treatment (T0) and after treatment (T1) were analyzed to quantify vertical skeletal and dentoalveolar changes. The study adhered to the Declaration of Helsinki and received approval from the Ethics Committee of the Policlinico of Bari (Prot. No. 971, Prot. 2427/CEL.; approved 1 October 2025; U.O. di Odontostomatologia). Written informed consent for treatment and data use was obtained from parents/legal guardians; age-appropriate assent was obtained from children.

### 2.2 Setting and participants

A total of nine growing patients (5 females, 4 males; mean age 9.8 ± 1.4 years) who met the inclusion criteria were included in the present case series. All subjects had complete pre- and post-treatment lateral cephalograms suitable for digital tracing. Among these, surface electromyographic (sEMG) analysis was successfully performed in six patients (66.7%), as three children did not achieve reliable muscle signal acquisition due to technical or compliance-related factors. Therefore, cephalometric outcomes refer to the entire sample (*n* = 9), while neuromuscular results are reported for the subgroup with valid EMG recordings (*n* = 6). Records were retrieved from the archives of the Department of Orthodontics, University Polyclinic of Bari (Italy). Nine consecutive growing patients with anterior open bite who had completed a defined AMCOP® “OPEN” protocol and had complete pre/post radiographic records were included.

#### 2.2.1 Inclusion criteria

- Growing patients in deciduous/mixed dentition with anterior open bite (overbite ≤ 0 mm) documented clinically and on T0 cephalogram.
- Treatment with AMCOP® “OPEN” elastodontic appliance as the primary modality.
- Availability of standardized lateral cephalograms at T0 and T1 suitable for digital tracing (Deltadent®).
- No adjunctive fixed appliances during the observation period.

#### 2.2.2 Exclusion criteria

- Previous orthodontic/orthopedic treatment affecting vertical dimensions.
- Craniofacial syndromes, cleft lip/palate, systemic conditions affecting growth.
- History of adeno-tonsillar surgery during the observation interval.
- Poor-quality radiographs precluding reliable landmark identification.

### 2.3 Intervention: AMCOP® “OPEN” protocol

All patients were treated with the AMCOP® “OPEN” elastodontic bioactivator [Micerium S.p.A.,Via G. Marconi,83 16036 Avegno (Ge) Italy], indicated for skeletal/dentoalveolar open bite patterns and characterized by a posteriorly raised occlusal plane and elastic flanges to promote mandibular counterclockwise rotation and anterior bite closure.

- Material & sizing: thermo-activatable polymer–elastomer (Shore 51/60); size selected by the inter-cuspidale width of upper first molars per manufacturer's chart.
- Therapeutic schedule: instructed 1 h/day + nocturnal wear, continuously for 6–8 months; thereafter night-time only until T1, depending on clinical stabilization.
- Adjuncts: no myofunctional exercises were systematically prescribed; standard hygiene and wear reinforcement were given at 4–6-week follow-ups.

### 2.4 Radiographs and cephalometric analysis

Standardized lateral cephalograms were obtained at T0 and T1 using department protocols (natural head position, maximum intercuspation, lips at rest). Digital tracings were performed in Deltadent® (Outside Format, Italy), which was also used to compute linear and angular measurements.

#### 2.4.1 Landmarks and planes

Landmark identification followed standardized cephalometric definitions as described in conventional orthodontic references. All landmarks were selected based on clear radiographic visibility and anatomical reproducibility to minimize identification errors. Reference planes were constructed consistently across all tracings using digitally assisted procedures provided by the Deltadent® software to ensure measurement standardization. Sella (S), Nasion (N), Point A (A), Point B (B), Anterior Nasal Spine (ANS), Posterior Nasal Spine (PNS), Gonion (Go), Gnathion (Gn), Menton (Me), and long axes of upper/lower incisors. Reference planes: SN, palatal plane (ANS–PNS), mandibular plane (Go–Gn), Frankfort (Po–Or), and occlusal plane.

#### 2.4.2 Primary outcomes (open-bite correction)

- Overbite (mm) (positive values indicate vertical overlap; ≤ 0 mm defines open bite).
- SN–GoGn (°) (mandibular plane to SN).
- PP–MP (ANS–PNS ^ Go–Gn,°) (intermaxillary divergence).
- ANS–Me (mm) (lower anterior facial height).

#### 2.4.3 Secondary outcomes

- FMA (°) (Frankfort–mandibular plane).
- Interincisal angle (°), U1–PP (°), L1–MP (°) (incisor inclinations).
- Overjet (mm) (to document associated sagittal/incisal changes).

All measurements were exported to an electronic spreadsheet for analysis.

### 2.5 Measurement reliability

A single calibrated examiner (orthodontist) performed all tracings. To assess intra-examiner reliability, 20% of radiographs (randomly selected, stratified by time point) were retraced after ≥7 days. We computed:

Intraclass correlation coefficients (ICC, two-way mixed, absolute agreement) for continuous variables.

Dahlberg's error (√*Σ*d²/2) for linear and angular measures. Intraclass correlation coefficients (ICC) were interpreted according to commonly accepted criteria (ICC ≥0.90 excellent agreement; 0.75–0.89 good; 0.50–0.74 moderate). An ICC threshold ≥0.80 was considered acceptable for inclusion of measurements in the final analysis. Dahlberg's error was calculated to quantify random measurement error, with values ≤0.5 mm for linear measurements and ≤0.5° for angular measurements considered clinically acceptable, in accordance with orthodontic reliability standards. When discrepancies exceeded these limits, tracings were reviewed and measurements repeated, and the meaning of the two closest values was retained.

### 2.6 Outcomes and time points

**Figure 1:**
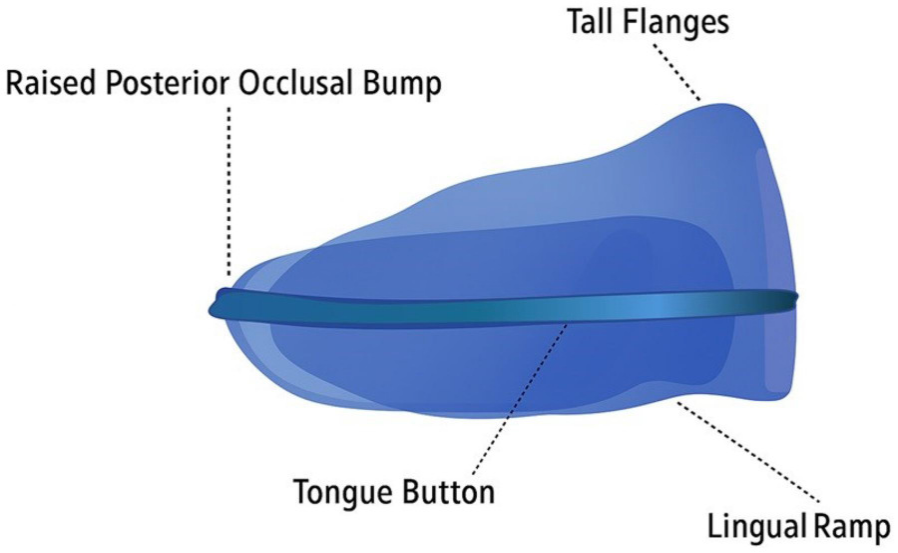
Amcop open device.

The primary endpoint was the change from T0 to T1 in overbite (mm) and vertical skeletal divergence (SN–GoGn, PP–MP). Secondary endpoints included changes in ANS–Me, FMA, incisor inclinations, and overjet. The observation interval corresponded to the active AMCOP® “OPEN” (Figure 1) phase plus stabilization until T1; exact durations are reported in the Results.

### 2.7 Cephalometric analysis

Cephalometric studies were conducted for each patient at the beginning of the treatment/observation period (T0) and at the conclusion of therapy (T1). The DeltaDent® 2.5.3 software was used for all cephalometric evaluations. Cephalometric dentoskeletal parameters and radiographic parameters related to airway dimensions were taken into account and then collected in a Microsoft Excel® spreadsheet (version 16.88) and subjected to statistical analysis. All the values obtained are presented in Table 1.

**Table 1:** Radiographic parameters.

Parameters	Definitions	Mean values (SD)
SNA	Angle between sella–nasion and nasion–point A segments.	82° (±2°)
SNB	Angle between sella–nasion and nasion–point B segments.	80° (±2°)
ANB	Angle between point A–nasion and nasion–point B segments.	2° (±2°)
Ans-Pns^Go-Gn	Intermaxillary angle, between bispinal plane (Ans-Pns) and mandibular plane (Go-Gn).	28° (±2°)
SN^Go-Gn	Mandibular angle between sella–nasion plane (S-N) and mandibular plane.	32° (±5°)
OVJ (mm)	Overjet, distance on the sagittal plane between the upper and lower incisors.	2.5 (±2.5 mm)
OVB (mm)	Overbite, distance on the vertical plane between the upper and lower incisors.	2.5 (±2.5 mm)
Wits		0 ± 2

We performed an electromyographic assessment of the masticatory muscles using a portable surface EMG system (**Teethan®**, Teethan S.p.A., Milan, Italy). All sEMG recordings were performed in a quiet room with subjects seated upright, head in natural position, feet flat on the floor, and hands resting on the thighs. Skin was cleansed with alcohol prior to electrode placement following SENIAM guidelines. Disposable pre-gelled surface electrodes were placed bilaterally on the anterior temporalis and masseter muscles along the muscle fibers. Each recording session consisted of two maximum voluntary clenches (MVC) on cotton rolls for signal normalization, followed by two MVCs performed in intercuspal position. Each contraction lasted 5 s, separated by 30-second rest intervals. Trials demonstrating motion artifacts or inconsistent activation patterns were discarded and repeated to ensure recording reliability. The EMG indices selected for analysis (POC TA/MM, BAR, TORS, ASIM, IMP) were chosen because they provide validated quantitative metrics of bilateral muscle symmetry, functional balance, occlusal load distribution, and overall neuromuscular efficiency, as previously described in standardized functional protocols using the Teethan® system. Bilateral surface EMG of the anterior temporalis and masseter muscles was recorded according to SENIAM recommendations. Recordings included two standardized maximum voluntary clenches (MVC) on cotton rolls for normalization, followed by two MVCs in intercuspal position. Signals were processed with the device software to compute EMG indices (POC TA/MM, BAR, TORS, IMP, ASIM, CL) and reported as pre- and post-treatment values (Teethan®, Teethan S.p.A., Via Forlanini, Garbagnate Milan, Italy).

## 3 Results

All 9 growing patients completed AMCOP® therapy (mean duration 14 ± 2 months) with excellent compliance and no adverse events. Cephalometric analysis revealed significant improvement in vertical and sagittal parameters. The mean overbite increased from 0.1 mm to 2.2 mm, indicating closure of anterior open bite. The SN–GoGn and PP–MP angles showed a mean reduction of 2.8° ± 1.9°, reflecting improved vertical control and slight counterclockwise mandibular rotation. The ANS–Me distance remained stable, confirming balanced lower facial height. Sagittal measurements demonstrated normalization of the ANB angle toward skeletal Class I (*Δ* ≈ –3.2°), while upper incisor inclination (U1–PP) decreased and the interincisal angle increased, suggesting better incisal guidance. Overjet was reduced in most cases, contributing to improved occlusal function and facial aesthetics. Surface electromyography (sEMG) indicated enhanced neuromuscular coordination: the barycenter (BAR) shifted toward midline, torsion (TORS) and asymmetry (ASIM) indices decreased, and the overall performance index (IMP) rose by an average of 40%. These findings confirm both morphological correction and functional rebalancing following AMCOP® therapy.

## 4 Case series

### 4.1 Case 1

**Figure 2:**
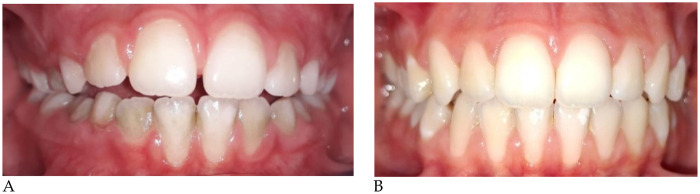
Pre **(A)** and post treatment **(B)** intraoral photos.

**Figure 3:**
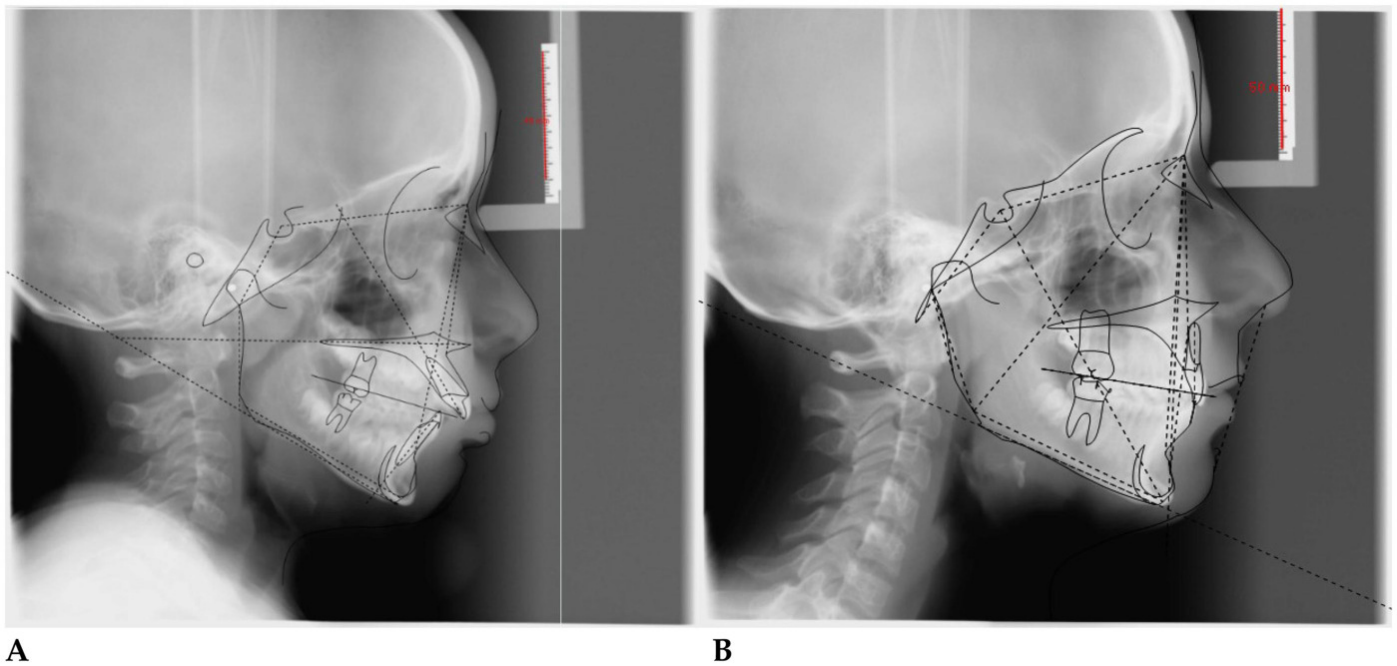
Initial **(A)** and final **(B)** cephalometry.

**C.A. (F) Years 8:** The patient underwent a 14-month orthodontic treatment using the *AMCOP® OPEN 3 (55 mm)* device. The following intraoral photographs (Figure 2) and cephalometric tracings (Figure 3) with relative pre- and post-treatment values in Table 2, show the pre-treatment and post-treatment conditions, highlighting the improvement in occlusal relationships and vertical dimensions.

**Table 2:** Comparative cephalometric values (pre- and post-treatment).

Parameter	Initial value	Final value	Reference value	Clinical comment
SNA (°)	75.2	74.5	82 ± 2	Persistent maxillary retrusion; slight improvement but upper jaw remains posteriorly positioned.
SNB (°)	71.3	69.7	80 ± 2	Increased mandibular retrusion; skeletal discrepancy remains significant.
ANB (°)	3.9	4.8	2 ± 2	Mild increase in sagittal discrepancy, maintaining skeletal Class II tendency.
S-N^Go-Gn (°)	37.3	42.1	32 ± 5	Worsening vertical pattern with mandibular clockwise rotation; skeletal open-bite tendency persists.
S-N^PO (°)	22.8	8.4	14 ± 3	Marked improvement in craniofacial inclination toward normodivergent pattern.
SN-Ba (°)	134.9	148.1	129 ± 5	Increased cranial base angle, consistent with posterior mandibular position.
IS/NA (°)	110.7	90.0	130 ± 5	Significant reduction in upper incisor proclination; better incisal control aiding bite closure.
II/NB (°)	94.1	81.5	94 ± 7	Normalization of lower incisor inclination; improved axial alignment.
Interincisal angle (°)	114.0	73.4	103 ± 2	Increased interincisal divergence, indicating improved incisor coordination post-therapy.
Mol Sup A.P. Occl (°)	82.4	93.5	90 ± 2	Improved molar angulation; mesial inclination suggests occlusal stabilization.
N-S-GoGn (°)	136.4	148.1	122 ± 5	Greater mandibular plane inclination; confirms vertical growth tendency.
Wits (mm)	1.0	0.9	0 ± 2	Stable sagittal skeletal relationship; slight improvement toward Class I.

#### 4.1.1 Final technical comment

The cephalometric analysis shows a reduction of the ANB angle (from 3.9° to 0.7°), indicating the improvement of the Class II skeletal discrepancy towards a more balanced relationship. An increase in the S-N^Go-Gn and S-N^Ba angle is also observed, suggesting a slight tendency towards postero-inferior rotation of the mandibular plane. The position of the upper incisors (IS/N-A) and lower incisors (II/N-B) shows a containment of vestibulisation, while the Wits value goes from +1.0 mm to −4.9 mm, confirming the achievement of a more neutral Class III relationship. Overall, the treatment resulted in an improvement of both the skeletal and the dento-alveolar component, with functional and aesthetic balance of the profile.

#### 4.1.2 Diagnostic treatment

The pre-treatment cephalometric analysis showed a Class II skeletal discrepancy characterised by upper maxillary (SNA = 75.2°) and mandibular (SNB = 71.3°) retrusion with an ANB angle = 3.9°, associated with hyperdivergence (S-N^Go-Gn = 37.3°) and upper incisor vestibuloposition (IS/N-A = 9.7 mm).

The facial profile was slightly protruded, with a tendency to postero-inferior rotation of the mandible and discrete dento-alveolar compensation.

#### 4.1.3 Evolution and response to treatment

Orthodontic treatment resulted in skeletal rebalancing:

- Reduction of the ANB angle from 3.9° to 0.7°, with improvement of the sagittal relationship.
- Slight increase in S-N^Go-Gn angle (from 37.3° to 39.4°), indicative of physiological mandibular adaptation.
- Containment of the superior incisive proclination and improved alignment of the lower incisors.
- Correction of the Wits value (from +1.0 mm to −4.9 mm), compatible with achieving a skeletal and dental Class I relationship.

##### 4.1.3.1 Prognosis

The long-term prognosis is favourable, as improvement in skeletal relationship has been achieved with satisfactory neuromuscular balance and occlusal stability. Periodic post-treatment monitoring (follow-up at 6 and 12 months) is recommended to check vertical stability and incisor inclination to prevent any functional or aesthetic recurrence.

### 4.2 Case 2

**Figure 4:**
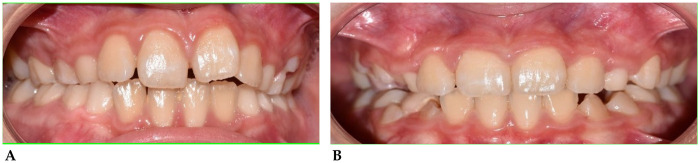
Frontal intraoral photo before **(A)** and after **(B)** treatment.

**Figure 5:**
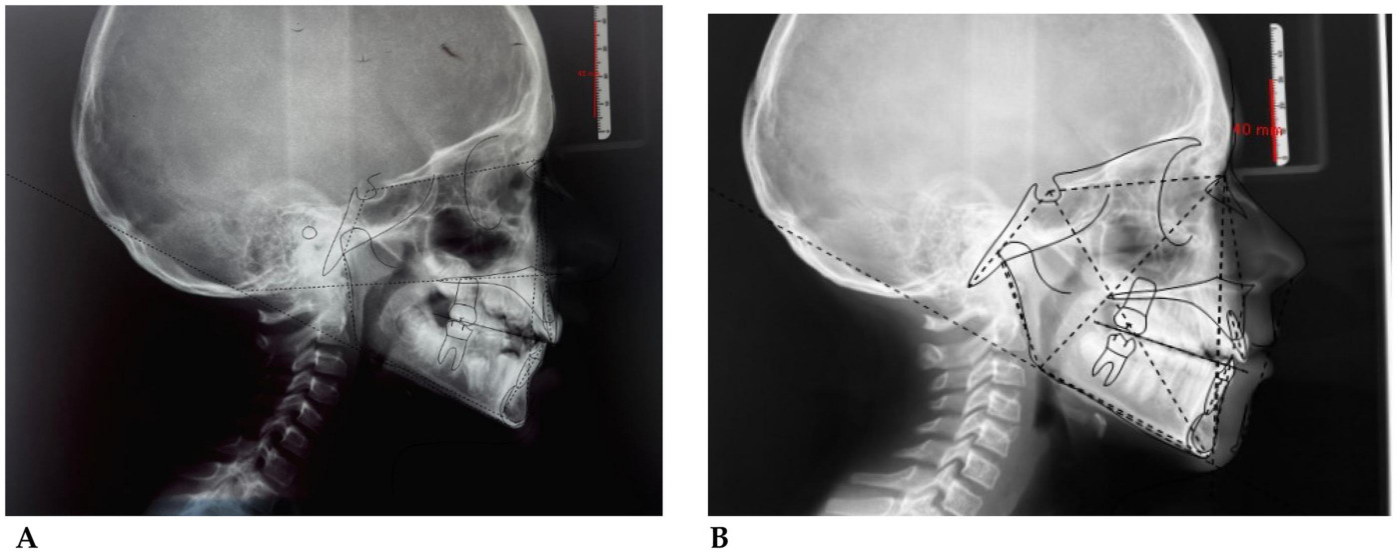
Initial **(A)** and final **(B)** cephalometry.

**B.S. (M) Years 9:** The patient underwent a 14-month orthodontic treatment using the *AMCOP®* Open 3**,** *(55 mm)* device**.** The following intraoral photographs (Figure 4) and cephalometric tracings (Figure 5) with relative pre- and post-treatment values in Table 3, show the pre-treatment and post-treatment conditions, highlighting the improvement in occlusal relationships and vertical dimensions.

**Table 3:** Comparative cephalometric values (Pre- and post-treatment).

Parameters	Pre-treatment value	Post-treatment value	Reference value	Comments
SNA (°)	∼82.1°	∼93.5°	82 ± 2	Marked increase, indicating greater forward position of the maxilla after treatment.
SNB (°)	∼76.0°	∼83.0°	80 ± 2	Also increased, showing mandibular advancement compared to baseline.
ANB (°)	∼6.1°	∼10.5°	2 ± 2	Larger difference between maxilla and mandible → persistence of skeletal Class II tendency.
SNA–SNP^Go-Gn (°) *(Maxillo-mandibular angle)*	∼29.1°	∼33.0°	20 ± 5	Increase suggests greater vertical divergence of the mandibular profile.
S-N^Go-Gn (°) *(Mandibular plane angle)*	∼36.2°	∼33.4°	32 ± 5	Slight reduction, but the angle remains moderately open — partial vertical control.
IS ∡ II (°) *(Upper–lower incisor angle)*	∼143.6°	∼140.3°	132 ± 6	Small reduction in interincisal angle, indicating better incisal coordination.
IS ∡ N-A (°) *(Upper incisor inclination)*	∼2.9°	∼–3.1°	22 ± 6	Retroclination of upper incisors after treatment, helping anterior bite closure.
II ∡ N-B (°) *(Lower incisor inclination)*	∼5.6°	∼5.3°	25 ± 7	Almost unchanged; lower incisor position remained stable.
Upper Molar ∡ Occlusal Plane (°)	∼93.9°	∼88.3°	90 ± 2	Decrease suggests improved molar inclination and occlusal leveling.
S–Co–Go (°)	∼148.1°	∼121.2°	143 ± 6	Notable reduction, consistent with decreased posterior mandibular rotation.
Co–Go–Gn (°)	∼126.8°	∼137.6°	120 ± 5	Increase indicates opening of the mandibular angle post-treatment.
Co–Go–N (°)	∼55.0°	∼62.6°	50 ± 2	Increased value reflects posterior mandibular displacement.
N–Go–Gn (°)	∼71.8°	∼75.0°	70 ± 2	Slight increase, showing mild clockwise mandibular rotation.
II^Go-Gn (°) *(Lower incisor–mandibular plane)*	∼94.7°	∼85.5°	93 ± 1	Decrease suggests uprighting of lower incisors.
S:N (°) *(Cranial base angle)*	∼83.9°	∼83.8°	74.5 ± 3	Practically unchanged, cranial base stability maintained.
Wits (mm)	∼–3.5	∼–6.6	0 ± 2	More negative value indicates persistence of skeletal Class II sagittal discrepancy.
IS^N–S (°) *(Upper incisor–cranial base angle)*	∼85.5°	∼100.8°	103 ± 2	Marked increase, showing improved upper incisor inclination and better anterior guidance.

#### 4.2.1 Final remarks—B.S.: General Observations on Open Bite

In open bite cases, an increase in posterior vertical dimensions, mandibular rotations, and/or divergence between the maxilla and mandible is often observed. The treatment appears to have produced significant changes in sagittal relationships (SNA, SNB, ANB) and mandibular angular parameters (Co-Go-Gn, Co-Go-N, N-Go-Gn), suggesting a restructuring of mandibular posture, with a likely rotation or alteration in the vertical dimension. The modification of incisal angles and the Wits appraisal indicates that the sagittal relationship between the upper and lower teeth has changed: the worsening of the Wits value (more negative) may reflect a sagittal shift of the lower dentition or a change in the alveolar reference point. The increase in the maxillo-mandibular angle (SNA-SNP^Go-Gn) suggests greater divergence after treatment, which could represent a potential issue if not properly controlled, as it tends to promote residual vertical opening.

### 4.3 Case 3

**Figure 6:**
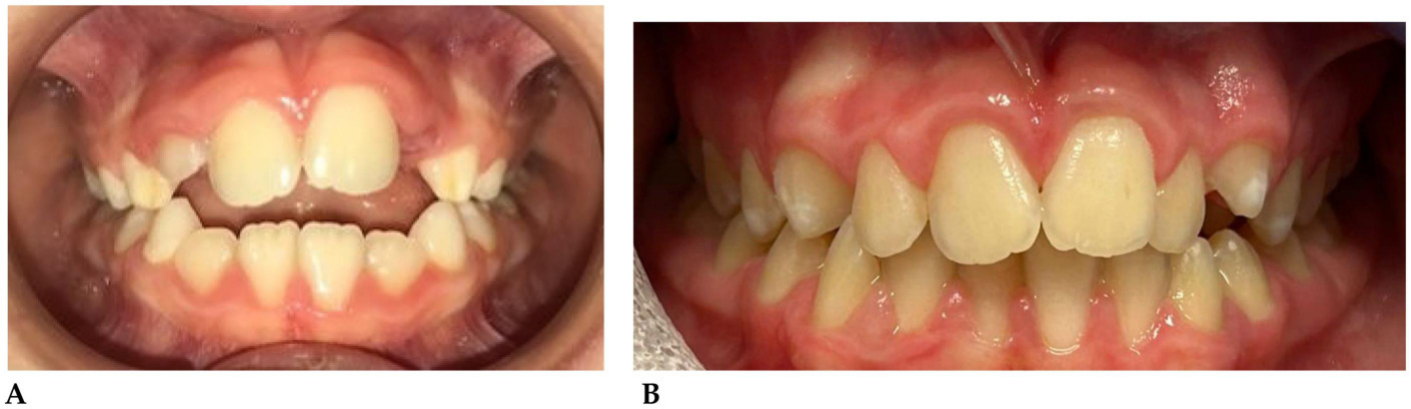
Frontal intraoral photo before **(A)** and after **(B)** treatment.

**Figure 7:**
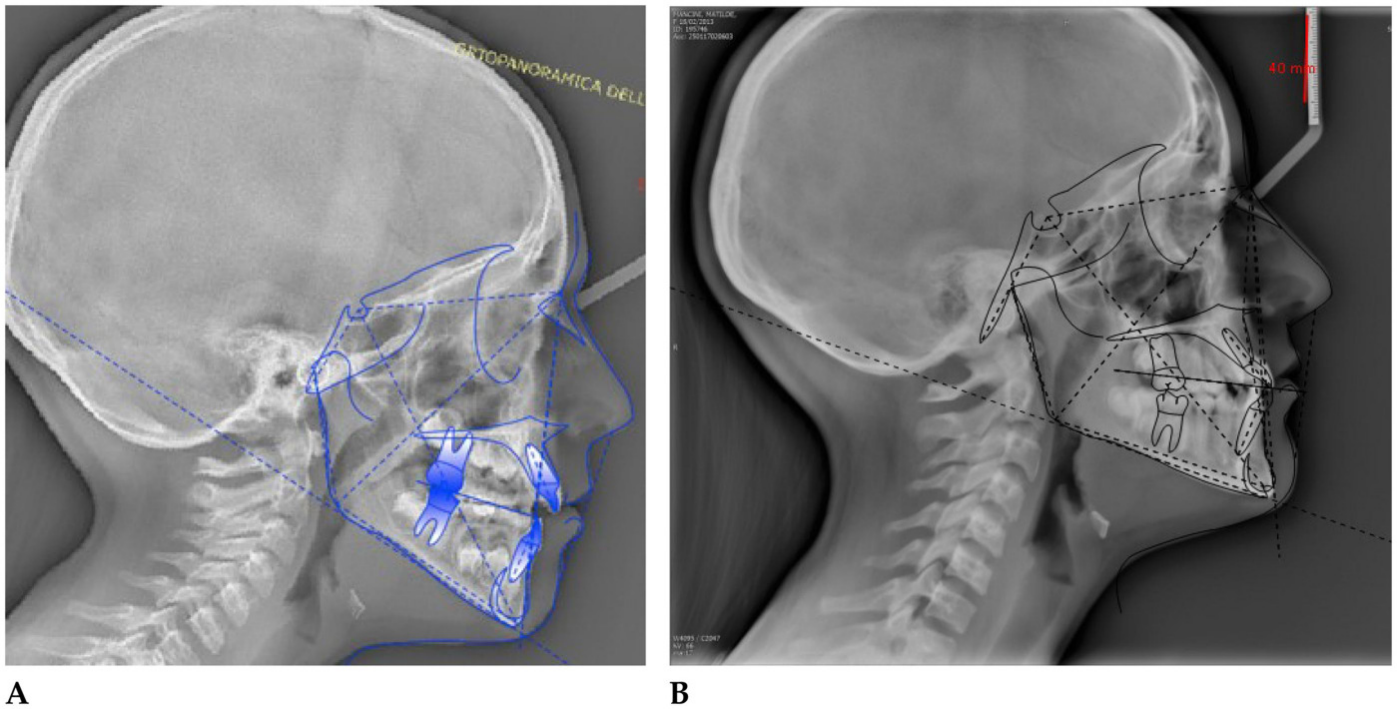
Initial **(A)** and final **(B)** cephalometry.

**M.M. (F) 8 anni e 2 mesi:** The patient underwent a 14-month orthodontic treatment using the *AMCOP®* Open 3, *(55 mm)* device. The following intraoral photographs (Figure 6) and cephalometric tracings with relative pre- and post-treatment cephalometric tracings (Figure 7) with relative pre- and post-treatment values in Table 4, show the pre-treatment and post-treatment conditions, highlighting the improvement in occlusal relationship and vertical dimension.

**Table 4:** Comparative cephalometric values (Pre- and post-treatment).

Parameter	Pre-treatment	Post-treatment	Normal range	Clinical comment
SNA (°)	87.2°	87.2°	82 ± 2	Slightly protrusive maxillary position, unchanged.
SNB (°)	77.1°	77.7°	80 ± 2	Persistent mandibular retrusion, slight improvement.
ANB (°)	10.1°	9.5°	2 ± 2	Persistent skeletal Class II discrepancy.
SNA–SNP∧Go–Gn (°)	23.2°	28.7°	20 ± 5	Increase in maxillo-mandibular angle; hyperdivergent trend linked to open bite.
S-N∧Go–Gn (°)	20.8°	20.9°	14 ± 3	Stable hyperdivergent angle confirms vertical growth tendency.
S-N∧Gn (°)	20.6°	20.0°	14 ± 3	Vertical mandibular growth pattern confirmed.
SN∧Ba (°)	135.5°	140.2°	129 ± 5	Slight posterior cranial rotation, consistent with open bite pattern.
IS∧SN (°)	87.5°	114.6°	76 ± 2	Marked labial inclination of upper incisors after treatment.
IS∧II (°)	131.2°	114.6°	130 ± 5	Reduction of interincisal angle, indicating flaring for bite closure.
II∧N-B (°)	9.7°	4.3°	4 ± 1	Normalization of lower incisor inclination.
II∧A-Pog (°)	5.9°	1.4°	2 ± 1	Slight retrusion of lower incisors, improving balance.
Upper Molar∧Occlusal Plane (°)	0.9°	110.9°	0 ± 1	Improved molar inclination toward a physiological position.
N-S∧CoP (°)	139.1°	137.3°	122 ± 5	Persistent posterior condylar position, not fully corrected.
S-Co-Go (°)	122.3°	133.3°	143 ± 6	Mandibular anterior rotation tendency, favorable for open bite closure.
Go-Me (°)	70.3°	85.3°	73 ± 1.5	Increase in mandibular height, consistent with post-treatment adaptation.
Wits (mm)	3.8	2.9	0 ± 2	Overall sagittal improvement, with mild residual Class II tendency.

#### 4.3.1 Final comment-open bite correction

Cephalometric analysis shows a marked positive evolution between start and end of treatment:

- Sagittal correction: change from skeletal class II (ANB 4.8°) to class I (ANB 0.8°), due to mandibular advancement and improved maxillary positioning.
- Vertical control: Reduction of the S-N^Go-Gn angle (from 42.1° to 38.5°) and normalisation of the palatine inclination (S-N^sna-snp from 8.4° to 1.2°) show mandibular anterior rotation and closure of the open bite.
- Incisive inclination: The upper and lower incisors underwent retrusion and verticalisation, contributing to bite closure and aesthetic improvement of the profile.
- Lip balance: the position of the lips in relation to the aesthetic line has returned within physiological limits, improving facial harmony.
- Wits from +1.0 to −5.8 mm indicate a more balanced sagittal relationship, compatible with a stable bite closure.

#### 4.3.2 Summary: open bite correction

Cephalometric analysis reveals significant improvement following treatment. The sagittal relationship was corrected from skeletal Class II to Class I through mandibular advancement and better maxillary positioning. Vertical control was achieved by reducing the mandibular plane angle and normalizing the palatal plane, indicating anterior mandibular rotation and closure of the open bite. Both upper and lower incisors were retruded and uprighted, contributing to bite closure and a more balanced facial profile. Lip position was also normalized, enhancing overall facial harmony.

### 4.4 Case 4

**Figure 8:**
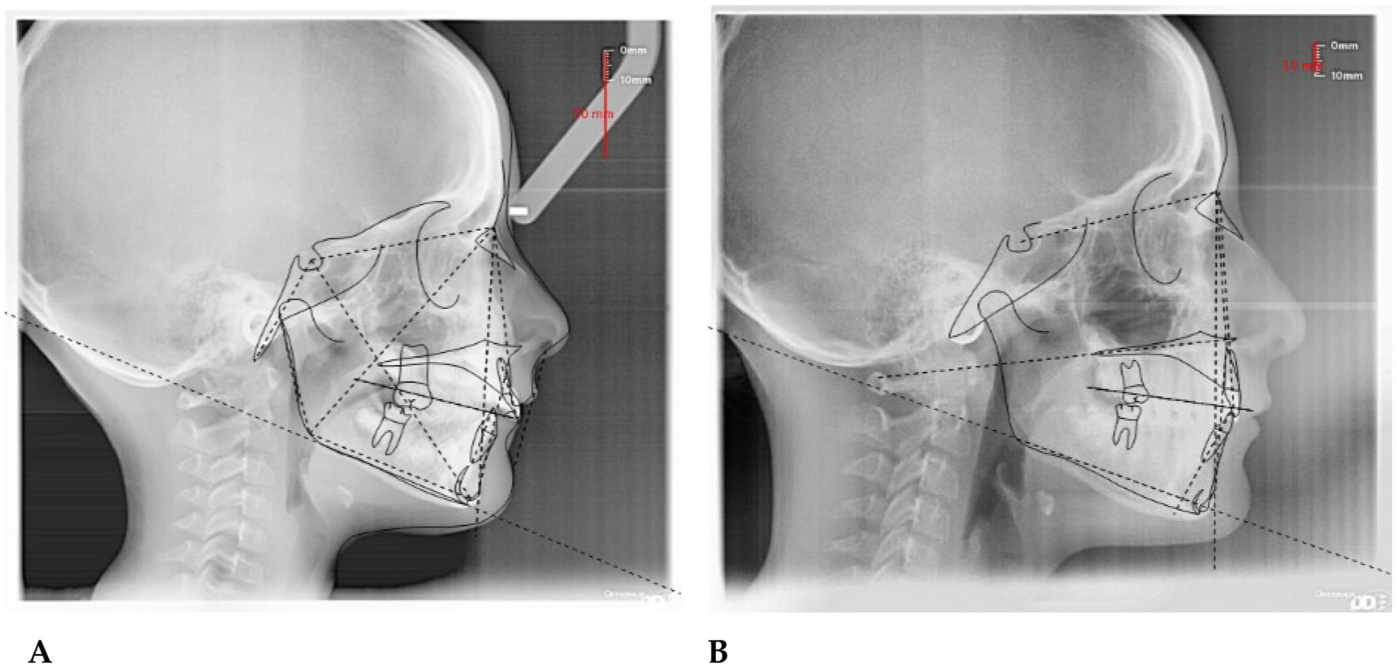
Initial **(A)** and final **(B)** cephalometry.

**Figure 9:**
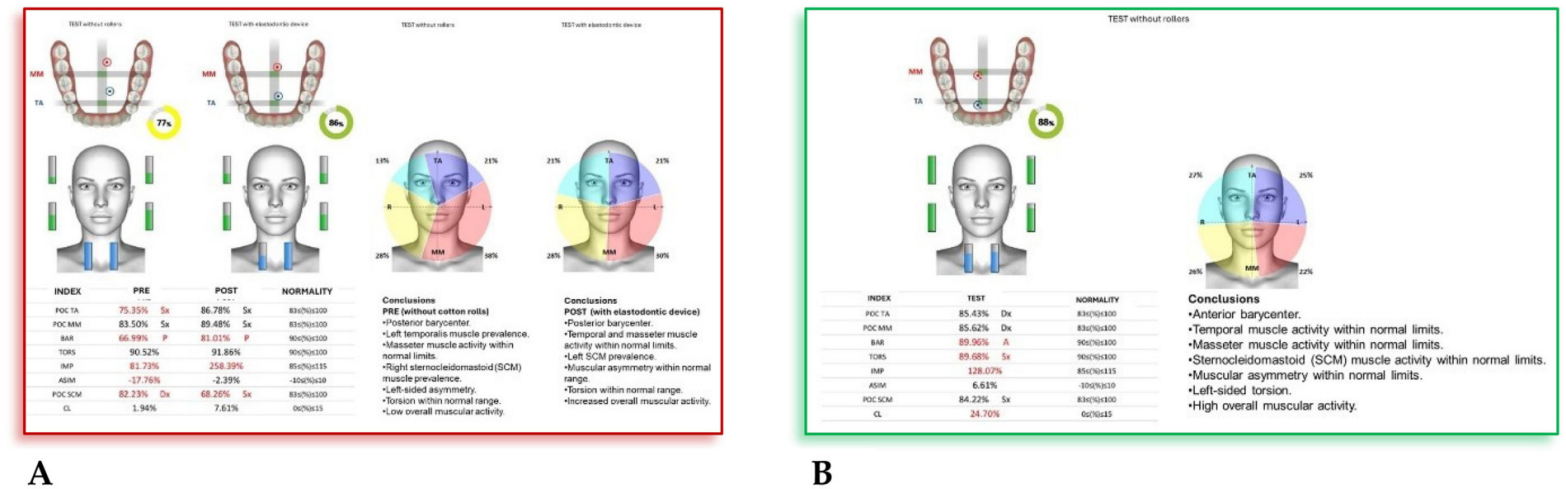
Surface electromyography (Teethan®) before **(A)** and after AMCOP® elastodontic therapy **(B)**. Post-treatment analysis shows normalization of the muscular barycenter, balanced activation of temporalis and masseter muscles, and reduction of asymmetry and torsional indices, confirming improved neuromuscular coordination and functional stability.

**P.M. (F) 10 years:** The patient underwent a 14-month orthodontic treatment using the *AMCOP®* Open 3**,** *(55 mm)* device**.** The following cephalometric tracing (Figure 8) with relative pre- and post-treatment values in Table 5, show the pre-treatment and post-treatment conditions, highlighting the improvement in occlusal relationships and vertical dimensions. Surface electromyography (Teethan®) (Figure 9) it was done before and after AMCOP® elastodontic therapy. Post-treatment analysis shows normalization of the muscular barycenter.

**Table 5:** Comparative cephalometric values (pre- and post-treatment).

Parameter	Initial value	Final value	Reference value	Clinical comment
SNA (°)	85.8	79.4	82 ± 2	Reduction of maxillary protrusion, contributing to improved bite closure.
SNB (°)	76.4	75.4	80 ± 2	Persistent mandibular retrusion, though without worsening the open bite.
ANB (°)	9.4	2.0	2 ± 2	Correction of skeletal relationship toward Class I; improved maxillo-mandibular balance.
SN-GoGn (°)	32.2	34.2	33 ± 2.5	Normodivergent vertical pattern maintained, with good control of vertical dimension.
+1/ANS-PNS (°)	87.5	87.5	110 ± 6	Reduced upper incisor inclination; contributed to anterior bite closure.
−1/Go-Gn (°)	99.2	99.2	94 ± 7	Appropriate lower incisor inclination, stable after treatment.
Interincisal angle (°)	143.0	147.2	132 ± 6	Increased interincisal angle, indicating better incisal control and reduction of open bite.
Overjet (mm)	3.0	1.4	3.5 ± 2.5	Decrease in overjet, showing improved incisal alignment.
Overbite (mm)	0.0	1.7	2.5 ± 2.5	Increase in overbite, consistent with closure of anterior open bite.
Wits (mm)	0.4	0.4	0 ± 2	Sagittal relationship remains overall balanced.

#### 4.4.1 Final clinical evaluation

Comparative cephalometric analysis revealed a marked improvement in both skeletal and dental relationships. Treatment resulted in normalization of the ANB angle, maintenance of the vertical growth pattern, and an increase in the interincisal angle. A reduction in overjet and an increase in overbite was observed, indicating closure of the anterior open bite. Upper incisor inclination remained well controlled, promoting occlusal and functional stability. Overall, the therapy led to significant harmonization of skeletal and dental components, with improvement in both oral function and facial aesthetics.

#### 4.4.2 Interpretation

Surface electromyography (Teethan®) revealed a clear improvement in neuromuscular coordination following AMCOP® elastodontic therapy. Before treatment, the patient exhibited a posterior barycenter, left temporalis prevalence, torsional imbalance, and low global muscle activity, suggesting altered functional recruitment.

After treatment, sEMG data demonstrated normalization of temporalis and masseter muscle activation, balanced SCM activity, correction of torsional asymmetry, and a shift toward anterior barycenter alignment with increased overall muscular efficiency. These findings indicate restored neuromuscular symmetry and improved functional integration of the stomatognathic system.

### 4.5 Case 5

**Figure 10:**
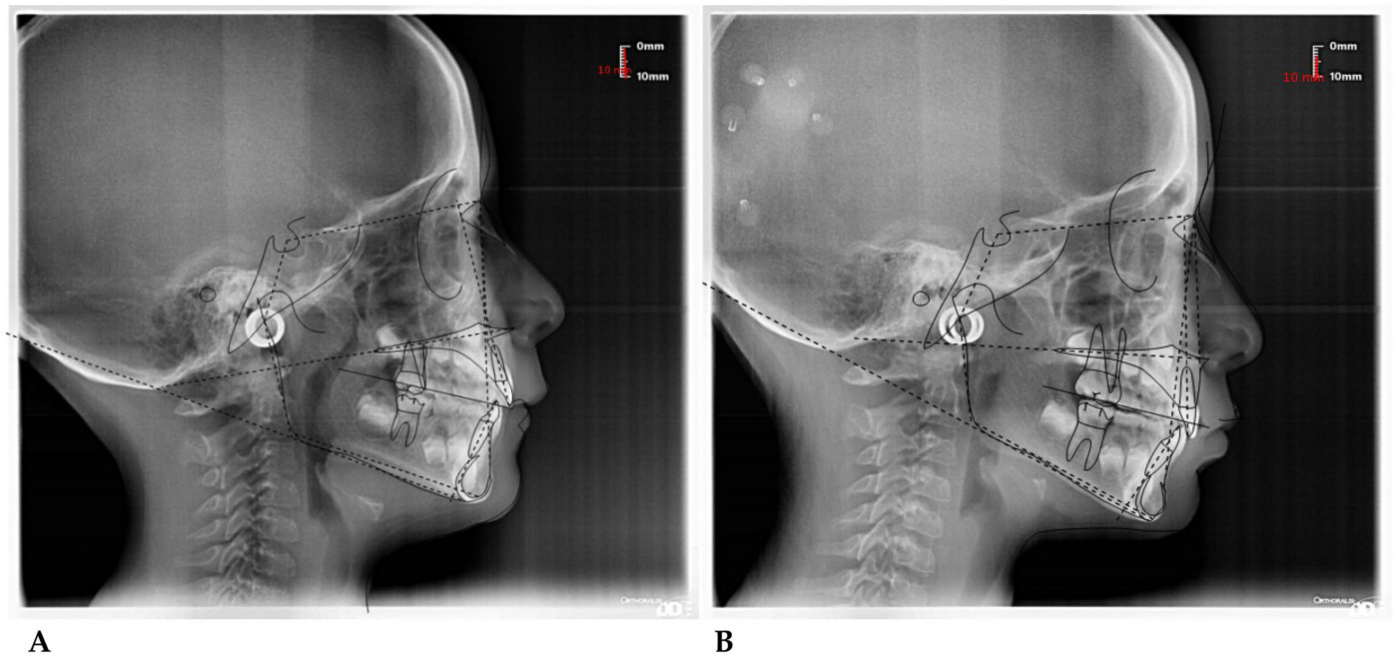
Initial **(A)** and final **(B)** cephalometry.

**Figure 11:**
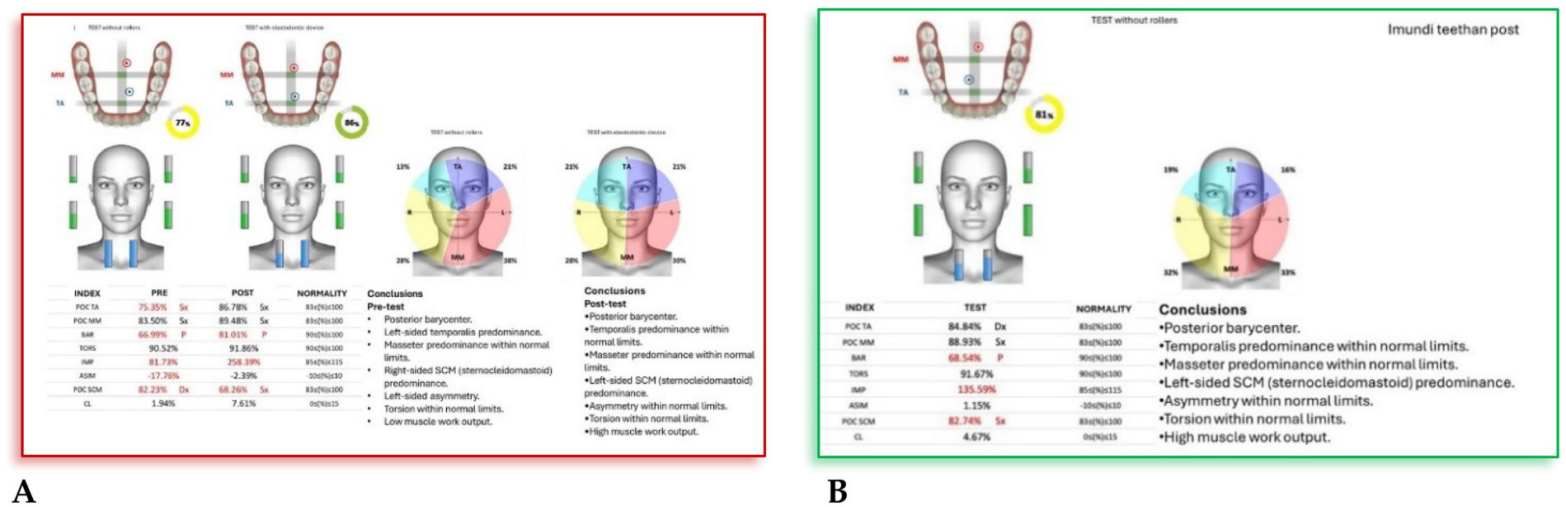
Electromyographic analysis with the Teethan® system. **(A)** Baseline assessment without elastomeric rollers and reevaluation following treatment, again without rollers; **(B)** final measurement during use of the elastodontic appliance. Comparisons between sessions reveal an overall increase in neuromuscular recruitment efficiency, reduced functional asymmetry, and a more centralized barycentric distribution, reflecting a more harmonious relationship between temporalis and masseter muscles.

**I.D. (F) 10 years:** The patient underwent a 14 months orthodontic treatment with AMCOP® 3- 55 mm device one hour per day plus every nigth. The following cephalometric tracings (Figure 10) with relative pre- and post-treatment values in Table 6, show the pre-trefollowing cephalometrict conditions, highlighting the improvement in occlusal relationships and vertical dimensions. Surface electromyography (Teethan®) (Figure 11) it was done before and after AMCOP® elastodontic therapy. Comparisons between sessions reveal an overall increase in neuromuscular recruitment efficiency, reduced functional asymmetry, and a more centralized barycentric distribution, reflecting a more harmonious relationship between temporalis and masseter muscles.

**Table 6:** Comparative cephalometric values (Pre- and post-treatment).

Measurement	Pre-treatment	Post-treatment	Normal range	Clinical comment
SNA (°)	81.2°	86.5°	82 ± 2	Improvement in maxillary position, indicating forward movement of the upper jaw.
SNB (°)	80.4°	78.1°	80 ± 2	Slight mandibular retrusion after treatment.
ANB (°)	0.8°	8.4°	2 ± 2	Shift from skeletal Class I to Class II relationship.
S-N^Go-Gn (°)	28.3°	25.1°	30 ± 5	Maintained hypodivergent pattern, indicating good vertical control.
S-N^SNA–SNP (°)	2.2°	8.9°	10 ± 3	Increase in palatal plane angle, suggesting improvement in vertical closure.
IS ∡ A (°)	6.1°	−2.5°	4 ± 1	Retroclination of upper incisors, contributing to closure of the anterior open bite.
II ∡ NB (°)	1.8°	2.2°	4 ± 1	Slight proclination of lower incisors, improving incisal contact.
Go–Me (°)	61.8°	86.3°	74 ± 5	Increase in mandibular height, indicating improved vertical dimension.
Wits (mm)	−10.5	0.0	0 ± 2	Correction of anteroposterior discrepancy, reflecting functional balance.
IS ∡ A–N–S (°)	92.5°	85.4°	103 ± 2	Reduction in upper incisor inclination, consistent with anterior bite closure.

#### 4.5.1 Final evaluation I.D

Cephalometric analysis shows an overall improvement in the anterior open bite. The treatment resulted in forward positioning of the maxilla (↑ SNA) and controlled vertical divergence (stable S-N^Go-Gn). Closure of the anterior open bite was achieved through retroclination of the upper incisors and slight proclination of the lower incisors, leading to improved incisal occlusion. The anteroposterior discrepancy was corrected (Wits from −10.5 to 0) while maintaining a harmonious facial profile. In summary, the treatment produced an effective correction of the open bite, with satisfactory skeletal and dental balance.

Teethan®-Based Surface EMG Analysis of Dental and Masticatory Muscle Activity (Figure 8).

#### 4.5.2 Interpretation

After 12 months of treatment with the AMCOP® Open appliance, the following improvements were documented:

- Notable transversal expansion: + 4.7 mm anteriorly and +6.2 mm posteriorly;
- Recovery of maxillary symmetry and proper intercuspidal coordination;
- Enhanced muscular coordination, with normalized POC values and a rise in global performance indices (IMP 135.6%);
- Barycenter stabilization with proportional activation between temporalis and masseter muscles.

Together, the sEMG results and morphological changes validate the efficacy of AMCOP® elastodontic therapy in optimizing both skeletal development and neuromuscular equilibrium during early mixed dentition.

### 4.6 Case 6

**Figure 12:**
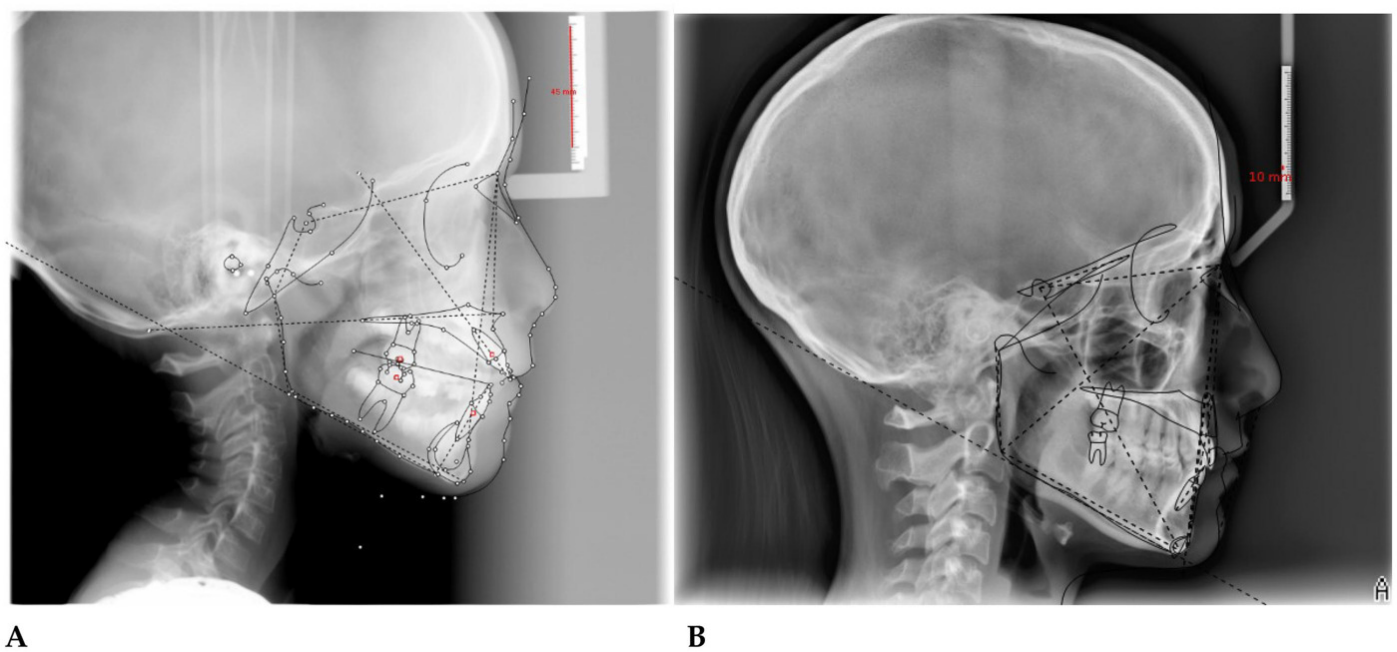
Initial **(A)** and final **(B)** cephalometry.

**Figure 13:**
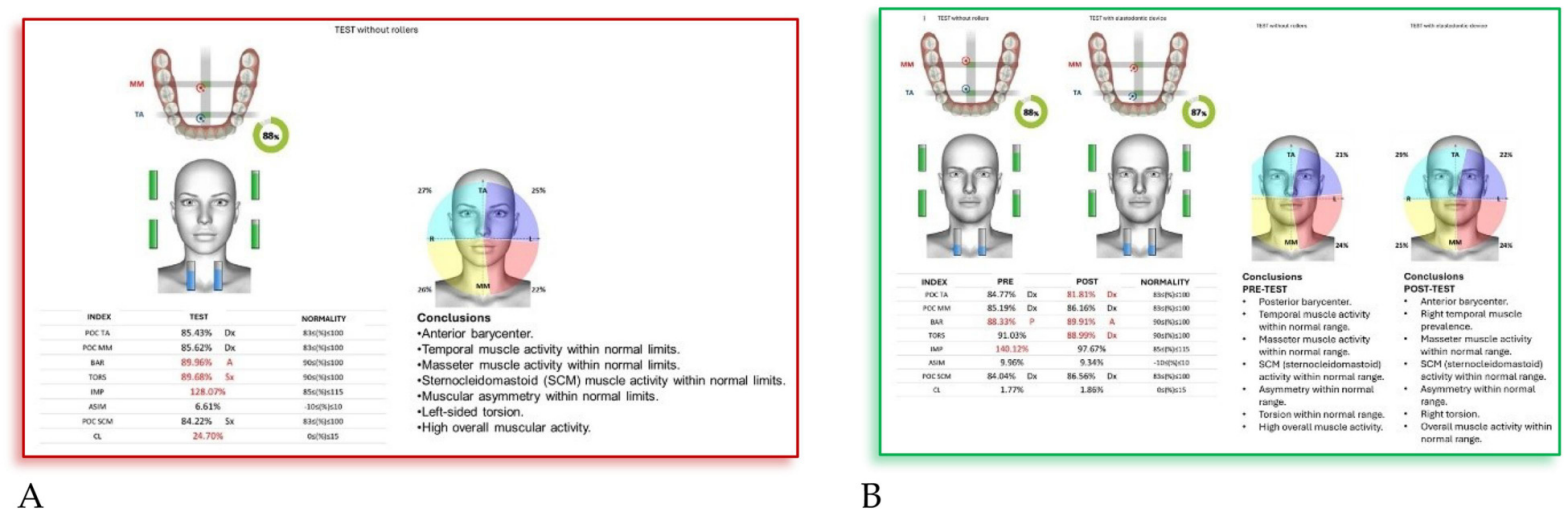
Surface electromyography (sEMG) neuromuscular assessment with Teethan® system performed before **(A)** and after **(B)** AMCOP® therapy with elastodontic activation. The color-coded distribution illustrates relative activity of TA, MM and SCM muscles; sagittal barycenter position, symmetry index and torsion pattern are also reported. Numerical indices reflect muscle recruitment balance and physiological neuromuscular coordination.

**B.V. (F) 8 years old:** The patient underwent a 14 months orthodontic treatment with AMCOP® 3- 55 mm device 1 h per day plus every night. The following cephalometric tracings (Figure 12) with relative pre- and post-treatment values in Table 7, show the pre-treatment and post-treatment conditions, highlighting the improvement in occlusal relationships and vertical dimensions. Surface electromyography (Teethan®) (Figure 13) it was done before and after AMCOP® elastodontic therapy. Comparisons between sessions reveal an overall increase in neuromuscular recruitment efficiency, reduced functional asymmetry, and a more centralized barycentric distribution, reflecting a more harmonious relationship between temporalis and masseter muscle.

**Table 7:** Comparative cephalometric values (pre- and post-treatment).

Parameter	Pre-treatment value	Post-treatment value	Reference value	Clinical comments
SNA (°)	∼82.1°	∼93.5°	82 ± 2	Marked increase, indicating greater forward position of the maxilla after treatment.
SNB (°)	∼76.0°	∼83.0°	80 ± 2	Also increased, showing mandibular advancement compared to baseline.
ANB (°)	∼6.1°	∼10.5°	2 ± 2	Larger difference between maxilla and mandible → persistence of skeletal Class II tendency.
SNA–SNP^Go-Gn (°) *(Maxillo-mandibular angle)*	∼29.1°	∼33.0°	20 ± 5	Increase suggests greater vertical divergence of the mandibular profile.
S-N^Go-Gn (°) *(Mandibular plane angle)*	∼36.2°	∼33.4°	32 ± 5	Slight reduction, but the angle remains moderately open — partial vertical control.
IS ∡ II (°) *(Upper–lower incisor angle)*	∼143.6°	∼140.3°	132 ± 6	Small reduction in interincisal angle, indicating better incisal coordination.
IS ∡ N-A (°) *(Upper incisor inclination)*	∼2.9°	∼–3.1°	22 ± 6	Retroclination of upper incisors after treatment, helping anterior bite closure.
II ∡ N-B (°) *(Lower incisor inclination)*	∼5.6°	∼5.3°	25 ± 7	Almost unchanged; lower incisor position remained stable.
Upper Molar ∡ Occlusal Plane (°)	∼93.9°	∼88.3°	90 ± 2	Decrease suggests improved molar inclination and occlusal leveling.
S–Co–Go (°)	∼148.1°	∼121.2°	143 ± 6	Notable reduction, consistent with decreased posterior mandibular rotation.
Co–Go–Gn (°)	∼126.8°	∼137.6°	120 ± 5	Increase indicates opening of the mandibular angle post-treatment.
Co–Go–N (°)	∼55.0°	∼62.6°	50 ± 2	Increased value reflects posterior mandibular displacement.
N–Go–Gn (°)	∼71.8°	∼75.0°	70 ± 2	Slight increase, showing mild clockwise mandibular rotation.
II^Go-Gn (°) *(Lower incisor–mandibular plane)*	∼94.7°	∼85.5°	93 ± 1	Decrease suggests uprighting of lower incisors.
S:N (°) *(Cranial base angle)*	∼83.9°	∼83.8°	74.5 ± 3	Practically unchanged, cranial base stability maintained.
Wits appraisal (mm)	∼–3.5	∼–6.6	0 ± 2	More negative value indicates persistence of skeletal Class II sagittal discrepancy.
IS^N–S (°) *(Upper incisor–cranial base angle)*	∼85.5°	∼100.8°	103 ± 2	Marked increase, showing improved upper incisor inclination and better anterior guidance.

#### 4.6.1 Clinical interpretation

At the beginning of treatment, the patient exhibited a skeletal Class II pattern, characterized by retrusion of both jaws, more pronounced in the mandible (SNA = 73.7°, SNB = 69.2°). The ANB angle of 4.5° confirmed a sagittal discrepancy between the maxilla and mandible, consistent with a Class II skeletal relationship.

Vertically, the S-N∧Go-Gn angle of 42.4° and the intermaxillary-mandibular plane angle (sna-snp∧Go-Gn = 30.5°) indicated a hyperdivergent facial pattern, typical of patients with anterior open bite and posterior mandibular rotation.

Dental findings, including a negative overbite (−4.5 mm) and an increased overjet (7 mm), confirmed the presence of an anterior open bite and upper incisor proclination, with poor anterior intercuspation and contact limited to posterior teeth.

#### 4.6.2 Post-Treatment changes

Following treatment, a remarkable improvement is observed in both the sagittal and vertical dimensions:

- The maxilla advanced by approximately 5° (SNA 79.1°).
- The mandible moved forward by nearly 7° (SNB 76.1°).
- The ANB angle decreased by 1.4°, indicating a transition toward a balanced Class I skeletal relationship.

Vertically, the mandibular plane angle decreased from 42.4° to 36.4°, showing a counter-clockwise rotation of the mandible and a closing of the occlusal plane—key changes for correcting an open bite.

Similarly, the intermaxillary-mandibular plane angle dropped from 30.5° to 21.9°, confirming excellent vertical control.

Although some dental parameters (e.g., IS∧AII and Wits post values) appear inconsistent and may require re-measurement, the overall trend demonstrates a harmonization of skeletal and dentoalveolar components.

The mandible rotated forward and upward, the anterior open bite closed, and the facial profile became more balanced, shifting from a dolichofacial to a near-mesofacial type.

#### 4.6.3 Post-Treatment open-bite findings

After treatment, patient Borgia demonstrated substantial improvement in the vertical dimension and a complete correction of the anterior open bite.

The mandibular plane angle (S-N∧Go-Gn) decreased markedly from 42.4° to 36.4°, indicating a counterclockwise rotation of the mandible and closure of the vertical dimension. This rotational change is one of the most important skeletal effects associated with open-bite correction, as it promotes forward and upward displacement of the chin and re-establishes incisal contact.

The intermaxillary-mandibular plane angle (Sna-Snp∧Go-Gn) showed a dramatic reduction from 30.5° to 21.9°, confirming excellent vertical control and a shift from a hyperdivergent to a mesofacial growth pattern. This demonstrates that the treatment effectively limited posterior vertical development and redirected mandibular growth anteriorly.

At the dento-alveolar level, the upper incisors—initially proclined with a negative overbite (−4.5 mm) and excessive overjet (7 mm)—were repositioned so that anterior intercuspation was restored and the bite fully closed.

The mandible's forward movement (SNB = 69.2° → 76.1°) and the slight maxillary advancement (SNA = 73.7° → 79.1°) improved the sagittal relationship and further contributed to vertical stability.

Functionally, these skeletal and dental modifications produced a stable anterior contact, an esthetically balanced lower facial third, and the elimination of the open-bite appearance.

The final cephalometric configuration describes a patient with Class I skeletal harmony, controlled vertical growth, and a physiological overbite, confirming that both the skeletal divergence and the dento-alveolar component of the open bite were successfully resolved.

#### 4.6.4 Interpretation

Following AMCOP® therapy with elastodontic elastics, a measurable neuromuscular improvement was observed.

Compared with the baseline condition, the post-treatment evaluation revealed:

- Transition from posterior to anterior neuromuscular barycenter, consistent with improved mandibular and head-neck posture.
- Normalization of masticatory muscle workload, with reduced hyperactivity and balanced recruitment of temporalis and masseter muscles.
- Stable SCM activation within normal physiological limits.
- Reduction in muscular asymmetry, indicating improved functional balance.
- Shift from left-sided torsion to right-sided mild physiological torsion, suggesting enhanced neuromuscular coordination.
- Overall reduction from elevated muscular effort to normal physiologic muscular activity, indicating improved efficiency and reduced functional stress.

These findings support that AMCOP® therapy, combined with elastodontic stimulation, may contribute to functional neuromuscular harmonization and balanced cranio-mandibular dynamics in growing patients.

### 4.7 Case 7

**Figure 14:**
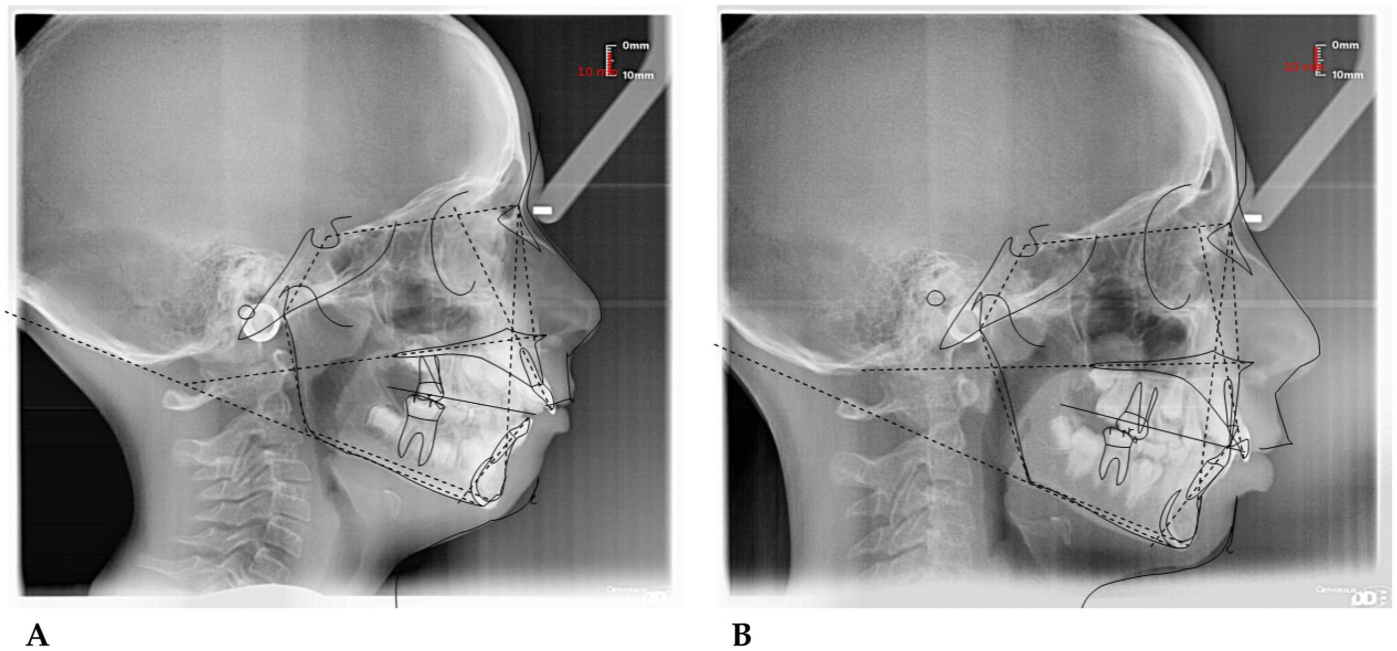
Initial **(A)** and final **(B)** cephalometry.

**Figure 15:**
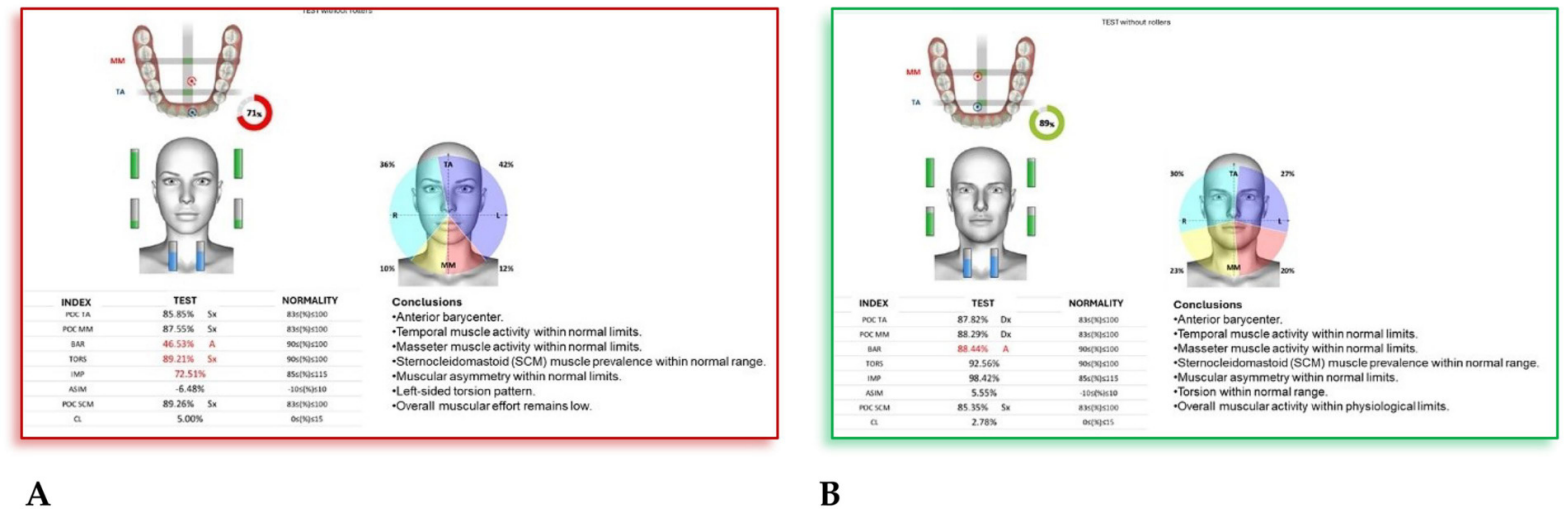
Surface electromyography (sEMG) neuromuscular assessment with Teethan® system performed before **(A)** and after **(B)** AMCOP® therapy with elastodontic activation. The color-coded distribution illustrates relative activity of TA, MM and SCM muscles; sagittal barycenter position, symmetry index and torsion pattern are also reported. Numerical indices reflect muscle recruitment balance and physiological neuromuscular coordination.

**F.F. (F) 11 Years old:** The patient underwent a orthodontic treatment with AMCOP® 3- 55 mm device 1 h per day plus every night, for a total of 10 months. The following cephalometric tracings (Figure 14) with relative pre- and post-treatment values in Table 8, show the pre-treatment and post-treatment conditions, highlighting the improvement in occlusal relationships and vertical dimensions. Surface electromyography (Teethan®) (Figure 15) it was done before and after AMCOP® elastodontic therapy. Post-treatment analysis shows normalization of the muscular barycenter.

**Table 8:** Comparative cephalometric values (pre- and post-treatment).

Parameter	Pre-treatment	Post-treatment	Normal range	Clinical comment
SNA (°)	87.2°	87.2°	82 ± 2	Slightly protrusive maxillary position, unchanged.
SNB (°)	77.1°	77.7°	80 ± 2	Persistent mandibular retrusion with slight improvement.
ANB (°)	10.1°	9.5°	2 ± 2	Persistent skeletal Class II discrepancy.
SNA–SNP∧Go-Gn (°)	23.2°	28.7°	20 ± 5	Increase in maxillo-mandibular angle, indicating hyperdivergent trend associated with open bite.
S-N∧Go-Gn (°)	20.8°	20.9°	14 ± 3	Stable hyperdivergent angle confirming vertical growth tendency.
S-N∧Gn (°)	20.6°	20.0°	14 ± 3	Vertical mandibular growth pattern confirmed.
SN∧Ba (°)	135.5°	140.2°	129 ± 5	Slight posterior cranial rotation, consistent with open bite pattern.
IS∧SN (°)	87.5°	114.6°	76 ± 2	Significant labial inclination of upper incisors after treatment.
IS∧II (°)	131.2°	114.6°	130 ± 5	Reduction of interincisal angle, indicating flaring for bite closure.
II∧N-B (°)	9.7°	4.3°	4 ± 1	Normalization of lower incisor inclination.
II∧A-Pog (°)	5.9°	1.4°	2 ± 1	Slight retrusion of lower incisors, contributing to balance.
Upper Molar∧Occlusal Plane (°)	0.9°	110.9°	0 ± 1	Improved molar inclination toward a more physiological position.
N-S∧CoP (°)	139.1°	137.3°	122 ± 5	Persistent posterior condylar position, not completely resolved.
S–Co–Go (°)	122.3°	133.3°	143 ± 6	Tendency toward mandibular anterior rotation, favorable for open bite closure.
Go–Me (°)	70.3°	85.3°	73 ± 1.5	Increase in mandibular height, consistent with functional post-treatment adaptation.
Wits (mm)	3.8	2.9	0 ± 2	Overall sagittal improvement with slight residual Class II tendency.

#### 4.7.1 Final technical comment

The comparative cephalometric analysis reveals a skeletal Class II pattern with slight improvement in mandibular position and a trend toward reduction of the anterior open bite.

The increase in the maxillo-mandibular angle and the greater inclination of the upper incisors indicate effective dentoalveolar compensation. Signs of hyperdivergence and a posteriorly positioned condyle persist; however, the tendency toward mandibular anterior rotation suggests a functionally favorable evolution.

Overall, treatment resulted in improved anterior intercuspation and enhanced facial aesthetics, despite a mild residual skeletal discrepancy.

#### 4.7.2 Interpretation

After AMCOP® therapy, surface electromyographic analysis revealed a clear improvement in neuromuscular balance.

Compared with the pre-treatment test, post-treatment data show:

- Restoration of barycenter symmetry and reduction of torsion (from 89.21% to within normal range);
- Normalization of BAR and IMP values, indicating improved overall muscle coordination and efficiency;
- Balanced activity of temporalis and masseter muscles, with asymmetry and torsion indices returning to physiological limits;
- Stabilization of global muscular function, as evidenced by normalized electromyographic indices.

These findings confirm the re-establishment of functional harmony between the right and left masticatory chains, supporting the effectiveness of AMCOP® elastodontic therapy in optimizing neuromuscular balance and occlusal stability.

### 4.8 Case 8

**Figure 16:**
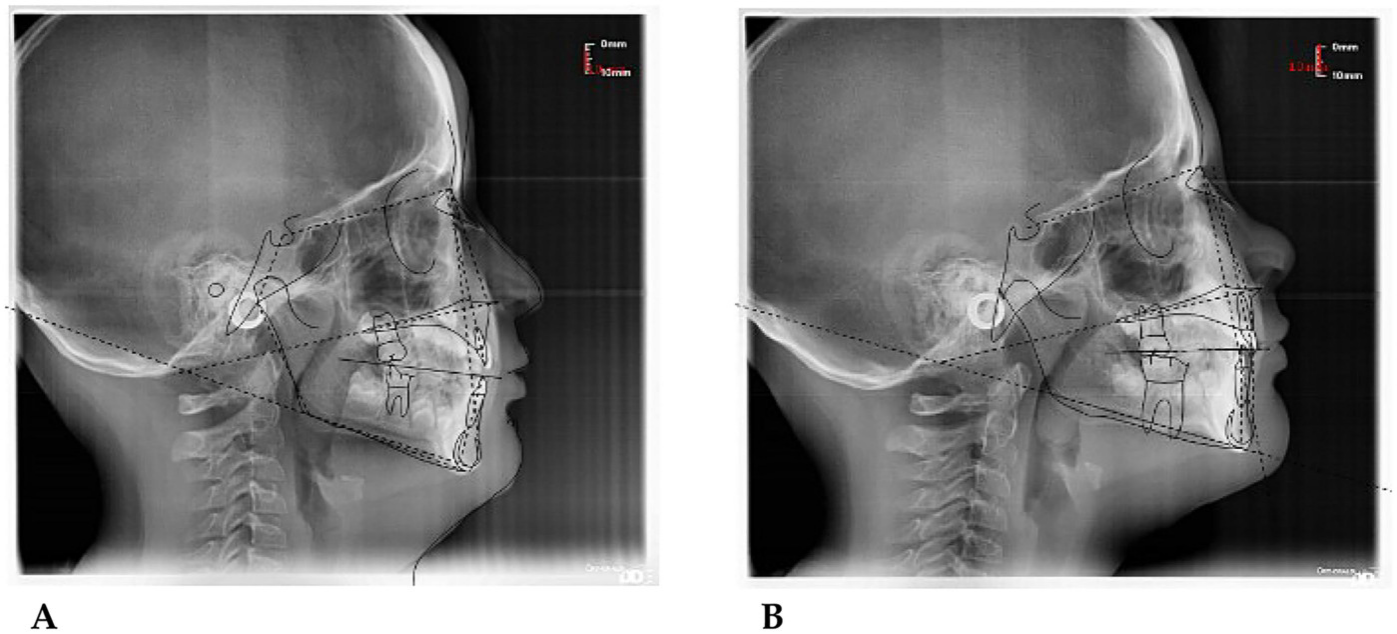
Initial **(A)** and final **(B)** cephalometry.

**Figure 17:**
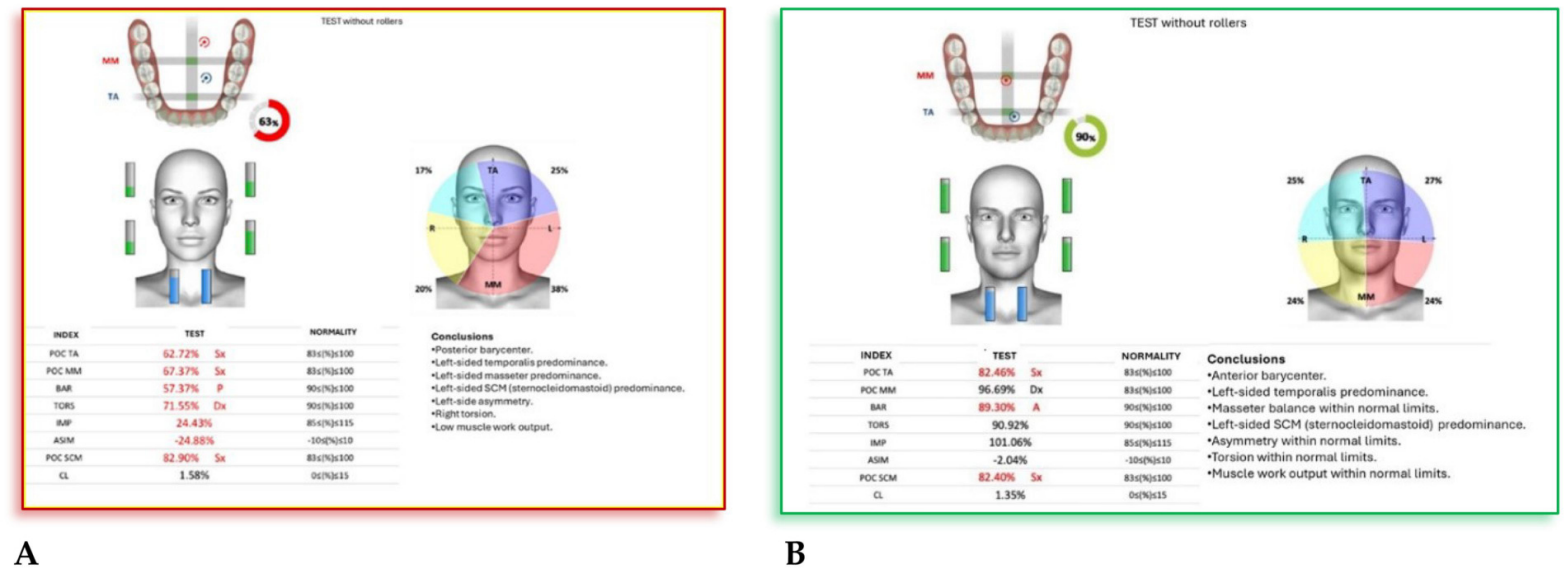
Surface electromyographic analysis using the Teethan® system [**(A)** before treatment; **(B)** after treatment].

**S.V. (M) 7 Years:** The patient underwent a orthodontic treatment with AMCOP® 3- 55 mm device 1 h per day plus every night, for a total of 10 months. The following cephalometric tracings (Figure 16) with relative pre- and post-treatment values in Table 9, show the pre-treatment and post-treatment conditions, highlighting the improvement in occlusal relationships and vertical dimensions. Surface electromyography (Teethan®) (Figure 17) it was done before and after AMCOP® elastodontic therapy. Post-treatment analysis shows normalization of the muscular barycenter.

**Table 9:** Comparative cephalometric values (pre- and post-treatment).

Parameter	Pre-treatment	Post-treatment	Reference value (normal range)	Interpretation
SNA (°)	92.4°	90.7°	82 ± 2	Slight reduction of maxillary protrusion; still mildly anterior to cranial base.
SNB (°)	80.8°	82.1°	80 ± 2	Forward repositioning of the mandible toward normal skeletal alignment.
ANB (°)	11.7°	8.6°	2 ± 2	Marked sagittal improvement from skeletal Class II toward Class I relationship.
S-N∧Go-Gn (°) *(Mandibular plane angle)*	30.5°	29.9°	33 ± 2.5	Vertical control maintained; transition from mild hyperdivergence to normodivergent pattern.
S-NBa (°) *(Cranial base angle)*	135.0°	—	131 ± 4	Normal cranial base morphology and inclination.
SND (°)	76.7°	—	76 ± 2	Stable mandibular position within normal range.
Upper incisor inclination (+1∧ANS-PNS) (°)	152.6°	89.0°	110 ± 6	Significant correction of upper incisor proclination; upright position achieved.
Lower incisor inclination (−1∧Go-Gn) (°)	86.9°	75.1°	94 ± 7	Marked retroclination of lower incisors; improved dental compensation.
Overjet (mm)	3.5	3.5	3.5 ± 2.5	Maintained within normal limits after alignment.
Overbite (mm)	3.3	3.3	2.5 ± 2.5	Normal vertical overlap preserved.
Interincisal angle (°)	—	166.9°	132 ± 6	Increased interincisal angle, consistent with incisor uprighting and improved esthetics.
Wits appraisal (mm)	−2.4	—	0 ± 2	Within normal range, indicating Class I skeletal balance.
IS–AN∧S (°) *(Upper incisor–cranial base angle)*	87.0°	—	103 ± 2	Within physiological limits; reflects normalized incisor inclination.
Facial pattern	Hyperdivergent tendency	Normodivergent	—	Vertical growth control achieved during treatment.
Final diagnosis	Skeletal Class II with upper incisor protrusion	Skeletal Class I with normal overjet/overbite and upright incisors	—	Harmonized skeletal and dental relationships; stable occlusal and aesthetic outcome.

#### 4.8.1 Clinical interpretation

At the beginning of treatment, patient Gianni presented a skeletal Class II relationship, with a protrusive maxilla (SNA = 92.4°) and a normal mandibular position (SNB = 80.8°). The ANB angle of 11.7° reflected a clear sagittal discrepancy between the jaws. Vertical parameters (S-N∧Go-Gn ≈ 30°) were within the normal range, suggesting a normodivergent facial pattern, not typically associated with open-bite tendencies.

The dentoalveolar analysis showed a marked proclination of upper incisors (IS∧AII = 152.6°) and lower incisor protrusion, producing a reduced interincisal angle and an aesthetic imbalance of the anterior segment.

This dental compensation contributed to the Class II appearance, even in the absence of vertical excess.

#### 4.8.2 Post-Treatment changes

After treatment, several favorable skeletal and dental modifications are evident:

- The maxilla shows a slight retrusion (SNA decreased by 1.7°),
- while the mandible advanced by approximately 1.3° (SNB = 82.1°),
- leading to a 3.1° reduction in the ANB angle and a transition toward a skeletal Class I relationship.

The vertical pattern remained stable (S-N∧Go-Gn ≈ 30°), confirming that vertical control was maintained throughout treatment. The most significant change occurred at the dentoalveolar level: the upper incisors were uprighted (89° vs. the pre-treatment 152° angle) and the lower incisors retroclined, resulting in a greater interincisal angle (166.9°) and improved anterior guidance. Overjet and overbite normalized at 3.5 mm, indicating stable and functional occlusion.

#### 4.8.3 Post-Treatment open bite findings

After treatment, the patient exhibits stable vertical control and a physiological overbite (3.3 mm).

The mandibular plane angle decreased slightly (−0.6°), suggesting a mild counterclockwise rotation of the mandible a favorable change that tends to close the bite anteriorly. The upper incisors were significantly uprighted (from 152.6° to 89°), which restored proper anterior contact and eliminated the pseudo-open bite component.

No evidence of vertical relapse or hyperdivergent tendency was detected; the patient maintained a normodivergent facial type with balanced lower facial height and functional incisal guidance.

**Teethan®-Based Surface EMG Analysis of Dental and Masticatory Muscle Activity**

Post-treatment recordings revealed a more centered barycentric distribution, improved coordination between temporalis and masseter muscles, and a noticeable decrease in asymmetry and torsional imbalances.

#### 4.8.4 Interpretation

After ten months of AMCOP® TC elastodontic therapy, the patient exhibited:

- A uniform and clinically relevant transverse expansion ranging from +4.5 to +4.9 mm across all evaluated regions;
- Elimination of the anterior crossbite tendency with enhanced transverse occlusal harmony;
- Rebalancing of neuromuscular activity, with normalization of POC, BAR, and TORS parameters.

Overall, the improvement in muscular coordination and interarch relationships confirms the orthopedic and functional effectiveness of AMCOP® Class III elastodontic treatment in promoting harmonious craniofacial growth.

### 4.9 Case 9

**Figure 18:**
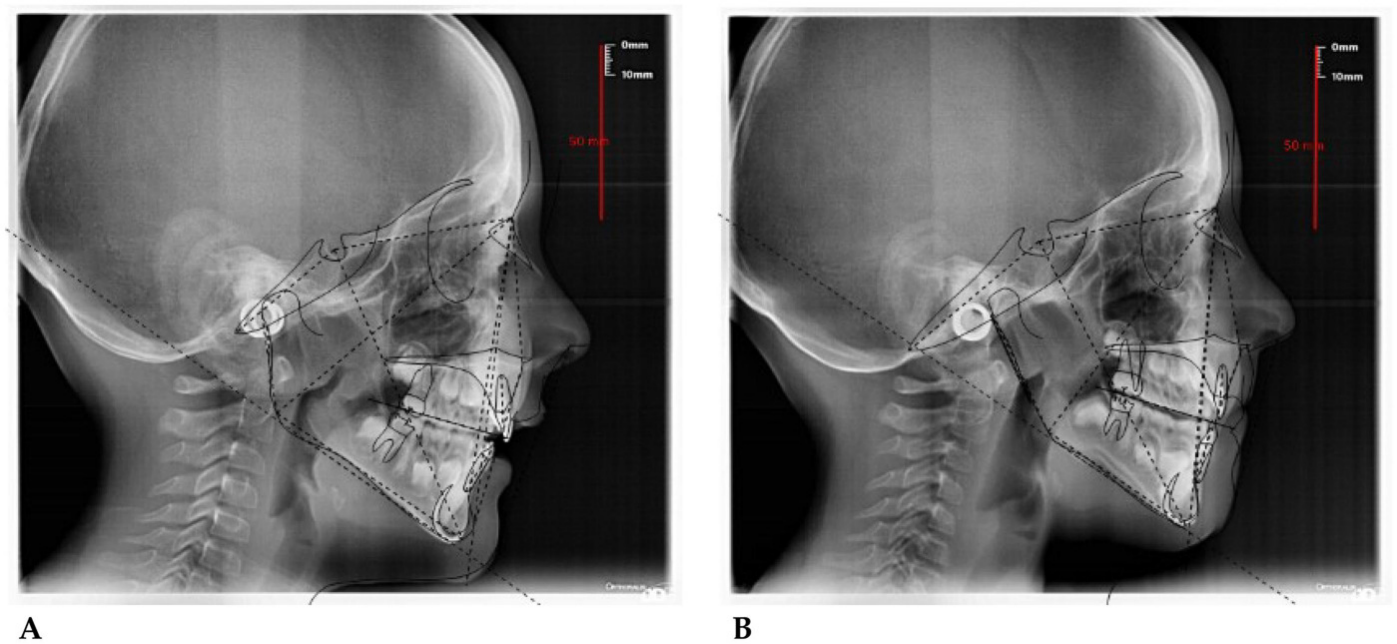
Initial **(A)** and final **(B)** cephalometry.

**Figure 19:**
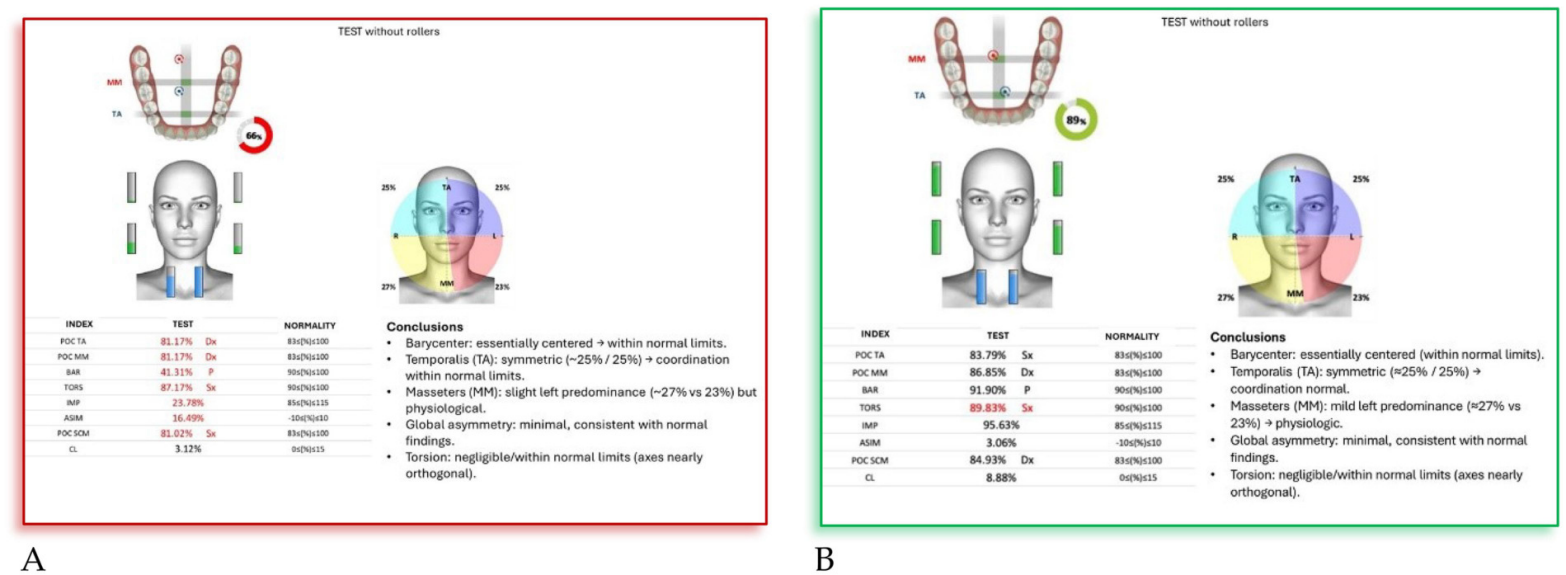
Teethan® surface electromyography. **(A)** Pre-treatment and **(B)** post-treatment.

**P.L. (M) 7 Years:** The patient underwent a 12-month orthodontic treatment using the *AMCOP®* Open 3**,** *(55 mm)* device**.** The following cephalometric tracings (Figure 18) with relative pre- and post-treatment values in Table 10, show the pre-treatment and post-treatment conditions, highlighting the improvement in occlusal relationships and vertical dimensions. Surface electromyography (Teethan®) (Figure 19) it was done before and after AMCOP® elastodontic therapy. Post-treatment analysis shows normalization of the muscular barycenter.

**Table 10:** Cephalometric comparison report: pre **(A)** and post **(B)** treatment.

Parameter	Pre-treatment	Post-treatment	Reference range	Clinical comment
SNA (°)	89.0	86.0	82 ± 2	Maxilla slightly protrusive initially, normalized post-treatment
SNB (°)	75.2	78.0	80 ± 2	Mandible retruded initially, improved after treatment
ANB (°)	13.8	8.0	2 ± 2	Skeletal Class II tendency reduced after treatment
SN-GoGn (°)	44.7	38.0	32 ± 5	Vertical growth pattern decreased, improving open bite tendency
SN-MP (°)	44.7	38.0	32 ± 5	Mandibular plane angle decreased, better vertical control
S-N^A (°)	89.0	86.0	82 ± 2	Upper jaw position normalized
S-N^B (°)	75.2	78.0	80 ± 2	Mandible advanced anteriorly
U1 to NA (°)	−5.4	21.0	22 ± 6	Upper incisors retroclined initially, uprighted post-treatment
L1 to NB (°)	3.9	20.0	25 ± 6	Lower incisors retroclined initially, improved alignment
Interincisal Angle (°)	171.7	135.0	130 ± 5	Severe incisor retroclination corrected
GoMe-SN (°)	44.5	38.5	32 ± 5	Improved mandibular plane angle—better bite closure
Wits (mm)	1.3	0.0	0 ± 2	Skeletal Class II improved toward normal

#### 4.9.1 Final comment

The cephalometric comparison shows significant improvement in the vertical and sagittal skeletal relationships. Initially, the patient exhibited a hyperdivergent skeletal pattern with mandibular retrusion and retroclined incisors, which contributed to an anterior open bite. Post-treatment, there is a clear reduction of the mandibular plane angle, forward positioning of the mandible, and normalization of incisor inclination. These changes indicate successful vertical control and bite closure, achieving functional and esthetic improvement consistent with open bite correction in an adult patient.

**Teethan®-Based Surface EMG Analysis of Dental and Masticatory Muscle Activity**

#### 4.9.2 Interpretation

After 12 months of treatment with the AMCOP® Class III elastodontic appliance, the patient showed:

- A clear transverse widening of the upper arch, particularly in the premolar region (+5.4 mm);
- Functional correction of anterior maxillary deficiency;
- Repositioning of the muscular barycenter and restoration of bilateral symmetry;
- A substantial increase in neuromuscular performance (IMP improved from 23.8% to 95.6%);
- Stable occlusal and muscular coordination, reflecting functional adaptation.

Overall, the findings confirm the effectiveness of early AMCOP® Class III therapy in promoting balanced transverse development and neuromuscular stabilization during growth.

## 5 Discussion

Early orthodontic intervention plays a crucial role in the effective management of malocclusions and associated orofacial dysfunctions (48, 109–112). A significant advancement in this field is represented by the development of Cranio-Occlusal-Postural Multifunctional Harmonizers (AMCOP® bioactivators) (4, 113–116). These appliances incorporate elastodontic principles that emphasize neuromuscular function to correct skeletal, dental, and muscular imbalances, thereby promoting ideal dental alignment and harmonious maxillary-mandibular growth (117–124). By improving tongue posture and swallowing function, AMCOP® bioactivators are particularly effective in the treatment of Class I, II, and III malocclusions, atypical swallowing, and related muscular dysfunctions (125–129). Their non-invasive design, combined with minimal discomfort and ease of use, ensures superior patient compliance compared with more conventional appliances such as Twin Blocks or Activators (130–133). These devices are suitable for both children and adults, as they reduce overall treatment time and provide flexibility throughout the various stages of therapy (60, 134–136 ). One of the major strengths of AMCOP® bioactivators lies in their ability to promote transverse development, achieved using a dedicated occlusal plane and a special elastomeric compound designed to act synergistically on skeletal structures, teeth, and musculature (137–147). In the presented cases, the clinical protocol initially involved the use of the AMCOP® Open appliance, which allowed targeted correction of transverse and vertical dimensions. This improvement was confirmed by comparative digital model analysis and cephalometric tracing, which documented the progression of therapy and the resolution of sagittal discrepancies (148–150). A common clinical error is to employ a Class-specific elastodontic appliance before addressing transverse discrepancies. In this protocol, the AMCOP® Open appliance was used for approximately nine months, followed by a Class-specific device to refine sagittal correction. The total treatment duration was approximately sixteen months. The appliance was worn for one hour during the day and passively throughout the night, without additional myofunctional exercises, since the act of swallowing itself provides sufficient and physiologically appropriate activation for therapeutic efficacy (62, 148–158). A major limitation of this study is the lack of a control group, which prevents definitive attribution of the observed changes exclusively to AMCOP® therapy. Since the sample consisted of growing patients, part of the improvements may reflect physiological craniofacial growth or spontaneous functional adaptation. Therefore, the present findings should be considered preliminary and hypothesis-generating. Controlled prospective studies are required to isolate treatment effects from growth-related changes.

## 6 Conclusions

AMCOP® elastodontic therapy was associated with clinically meaningful improvements in vertical skeletal relationships and neuromuscular coordination in a pediatric population treated during active growth. Cephalometric analysis showed closure or reduction of anterior open bite, improved control of mandibular plane inclination, normalization of intermaxillary divergence, and more physiological incisor inclinations with improved overjet/overbite. Parallel sEMG findings, including normalization of the functional barycenter, reduction of torsion and asymmetry, and increased impact/efficiency indices, suggest a favorable neuromuscular rebalancing rather than purely dentoalveolar camouflage. The staged protocol (initial Open phase to recover transverse and vertical corridors, followed by class-oriented refinement when indicated) proved well tolerated, minimally invasive, and compatible with high compliance. No relevant adverse events were recorded. However, this work represents a preliminary pilot case series with a small sample size and a retrospective single-arm design. Therefore, conclusions must be interpreted with caution, and no definitive causal inference can be drawn. From a clinical perspective, these preliminary findings support the potential role of early interceptive treatment integrating orthopedic guidance and neuromuscular rehabilitation in selected growing patients, particularly those at risk of vertical relapse. Future prospective controlled trials with larger samples and long-term follow-up are warranted to confirm treatment effectiveness and stability. Nevertheless, the retrospective single-arm design and the absence of an untreated or alternative-appliance control group represent a major limitation that substantially limits causal inference.

## Data Availability

The original contributions presented in the study are included in the article/Supplementary Material, further inquiries can be directed to the corresponding author/s.

## Glossary

AMCOP®: Armonizzatori Multifunzionali Cranio-Occluso-Posturali
Deltadent®: Digital Cephalometric Software
sEMG: Surface Electromyography
POC: Percentage of Overlapping Coefficient
TA: Temporalis Anterior
MM: Masseter Muscle
BAR: Barycenter Index
TORS: Torsion Index
IMP: Impact Index
ASIM: Asymmetry Index
CL: Clenching Level
SENIAM: Surface Electromyography for the Non-Invasive Assessment of Muscles
ICC: Intraclass Correlation Coefficient
ANS: Anterior Nasal Spine
PNS: Posterior Nasal Spine
S: Sella
N: Nasion
A: Point A (Subspinale)
B: Point B (Supramentale)
Go: Gonion
Gn: Gnathion
Me: Menton
Pog: Pogonion
SNA (°): Sella–Nasion–Point A angle
SNB (°): Sella–Nasion–Point B angle
ANB (°): Point A–Nasion–Point B angle
SN–GoGn (°): Sella–Nasion to Gonion–Gnathion angle
PP–MP (°): Palatal Plane to Mandibular Plane angle
ANS–Me (mm): Anterior Nasal Spine to Menton
FMA (°): Frankfort–Mandibular Plane Angle
U1–PP (°): Upper incisor to Palatal Plane
L1–MP (°): Lower incisor to Mandibular Plane
IS: Upper Incisor Axis
II: Lower Incisor Axis
IS∧II (°): Interincisal Angle
OVJ (mm): Overjet
OVB (mm): Overbite
Wits (mm): Wits Appraisal
SN∧Ba (°): Sella–Nasion to Basion angle
SNA–SNP∧Go–Gn (°): Maxillo–Mandibular Angle
Go–Me (°): Gonion–Menton angle
SND (°): Sella–Nasion–D point angle
N–S∧CoP (°): Nasion–Sella to Condylar Point
S–Co–Go (°): Sella–Condylion–Gonion angle
Co–Go–Gn (°): Condylion–Gonion–Gnathion angle
Go–Me–SN (°): Gonion–Menton to Sella–Nasion
T0/T1: Time 0/Time 1
MVC: Maximum Voluntary Clench
SD: Standard Deviation
*Δ* (Delta): Change/Difference
*α* (Alpha): Significance Level

## References

[1] Fields HW Proffit WR Nixon WL Phillips C Stanek E. Facial pattern differences in long-faced children and adults. Am J Orthod. (1984) 85:217–23. 10.1016/0002-9416(84)90061-7 6608274

[2] Nanda SK. Patterns of vertical growth in the face. Am J Orthod Dentofacial Orthop. (1988) 93:103–16. 10.1016/0889-5406(88)90287-9 3422525

[3] Nahoum HI. Vertical proportions: a guide for prognosis and treatment in anterior open-bite. Am J Orthod. (1977) 72:128–46. 10.1016/0002-9416(77)90055-0 268145

[4] Subtelny JD Sakuda M. Open-Bite: diagnosis and treatment. Am J Orthod. (1964) 50:337–58. 10.1016/0002-9416(64)90175-7

[5] Sassouni V. A classification of skeletal facial types. Am J Orthod. (1969) 55:109–23. 10.1016/0002-9416(69)90122-5 5249177

[6] Siriwat PP Jarabak JR. Malocclusion and facial morphology is there a relationship? An epidemiologic study. Angle Orthod. (1985) 55:127–38. 10.1043/0003-3219(1985)055/3C0127:MAFMIT/3E2.0.CO;2 3874569

[7] Cangialosi TJ. Skeletal morphologic features of anterior open bite. Am J Orthod. (1984) 85:28–36. 10.1016/0002-9416(84)90120-9 6581725

[8] Sassouni V Nanda S. Analysis of dentofacial vertical proportions. Am J Orthod. (1964) 50:801–23. 10.1016/0002-9416(64)90039-9

[9] Simion M Rocchietta I Kim D Nevins M Fiorellini J. Vertical ridge augmentation by means of deproteinized bovine bone block and recombinant human platelet-derived growth factor-BB: a histologic study in a dog model. Int J Periodontics Restorative Dent. (2006) 26:415–23. 17073351

[10] Inchingolo R Maino C Gatti M Tricarico E Nardella M Grazioli L Gadoxetic acid magnetic-enhanced resonance imaging in the diagnosis of cholangiocarcinoma. World J Gastroenterol. (2020) 26:4261–71. 10.3748/wjg.v26.i29.4261 32848332 PMC7422539

[11] Arulselvan P Fard MT Tan WS Gothai S Fakurazi S Norhaizan ME Role of antioxidants and natural products in inflammation. Oxid Med Cell Longev. (2016) 2016:5276130. 10.1155/2016/5276130 27803762 PMC5075620

[12] Malcangi G Patano A Morolla R De Santis M Piras F Settanni V Analysis of dental enamel remineralization: a systematic review of technique comparisons. Bioengineering. (2023) 10:472. 10.3390/bioengineering10040472 37106659 PMC10135549

[13] Coloccia G Inchingolo AD Inchingolo AM Malcangi G Montenegro V Patano A Effectiveness of dental and maxillary transverse changes in tooth-borne, bone-borne, and hybrid palatal expansion through cone-beam tomography: a systematic review of the literature. Medicina. (2021) 57:288. 10.3390/medicina57030288 33808680 PMC8003431

[14] Vaid NR Sabouni W Wilmes B Bichu YM Thakkar DP Adel SM. Customized adjuncts with clear aligner therapy: “the golden circle model” explained!. J World Fed Orthod. (2022) 11:216–25. 10.1016/j.ejwf.2022.10.005 36400659

[15] Sabouni W Muthuswamy Pandian S Vaid NR Adel SM. Distalization using efficient attachment protocol in clear aligner therapy—a case report. Clin Case Rep. (2023) 11:e6854. 10.1002/ccr3.6854 36698525 PMC9860201

[16] Yan X Zhang X Ren L Yang Y Wang Q Gao Y Effectiveness of clear aligners in achieving proclination and intrusion of incisors among class II division 2 patients: a multivariate analysis. Prog Orthod. (2023) 24:12. 10.1186/s40510-023-00463-6 37009943 PMC10068686

[17] Patel DN. Invisalign Case Study Part One: The Deep Bite—dentistry Online. Dentistry.co.uk (2021).

[18] Cui J-Y Ting L Cao Y-X Sun D-X Bing L Wu X-P. Morphology changes of maxillary molar distalization by clear aligner therapy. Int J Morphol. (2022) 40:920–6. 10.4067/S0717-95022022000400920

[19] D’Antò V Valletta R Ferretti R Bucci R Kirlis R Rongo R. Predictability of maxillary molar distalization and derotation with clear aligners: a prospective study. Int J Environ Res Public Health. (2023) 20:2941. 10.3390/ijerph20042941 36833638 PMC9957205

[20] Erdinç AE Ugur T Erbay E. A comparison of different treatment techniques for posterior crossbite in the mixed dentition. Am J Orthod Dentofacial Orthop. (1999) 116:287–300. 10.1016/s0889-5406(99)70240-4 10474101

[21] Halicioğlu K Yavuz İ. A comparison of the sagittal and vertical dentofacial effects of maxillary expansion produced by a memory screw and a hyrax screw. Aust Orthod J. (2016) 32:31–40. 10.21307/aoj-2020-110 27468589

[22] Manzella KS Warunek S Conley RS Al-Jewair T. A controlled clinical study of the effects of the Ni-Ti Memoria® leaf spring activated expander. Aust Orthod J. (2018) 34:196–204. 10.21307/aoj-2020-071

[23] Ugolini A Cossellu G Farronato M Silvestrini-Biavati A Lanteri V. A multicenter, prospective, randomized trial of pain and discomfort during maxillary expansion: leaf expander versus hyrax expander. Int J Paediatr Dent. (2020) 30:421–8. 10.1111/ipd.12612 31894603

[24] Bavetta G Bavetta G Randazzo V Cavataio A Paderni C Grassia V A retrospective study on insertion torque and implant stability quotient (ISQ) as stability parameters for immediate loading of implants in fresh extraction sockets. Biomed Res Int. (2019) 2019:9720419. 10.1155/2019/9720419 31781659 PMC6875416

[25] Timms DJ. A study of basal movement with rapid maxillary expansion. Am J Orthod. (1980) 77:500–7. 10.1016/0002-9416(80)90129-3 6989258

[26] Baccetti T Franchi L McNamara JA. An improved version of the cervical vertebral maturation (CVM) method for the assessment of mandibular growth. Angle Orthod. (2002) 72:316–23. 10.1043/0003-3219(2002)072/3C0316:AIVOTC/3E2.0.CO;2 12169031

[27] d'Apuzzo F Nucci L Strangio BM Inchingolo AD Dipalma G Minervini G Applied Sciences | Free Full-Text | Dento-Skeletal Class III Treatment with Mixed Anchored Palatal Expander: A Systematic Review. Available online at: https://www.mdpi.com/2076-3417/12/9/4646 (Accessed June 27, 2023).

[28] Inchingolo F Tatullo M Abenavoli FM Marrelli M Inchingolo AD Corelli R Surgical treatment of depressed scar: a simple technique. Int J Med Sci. (2011) 8:377–9. 10.7150/ijms.8.377 21698056 PMC3119380

[29] Inchingolo F Tatullo M Pacifici A Gargari M Inchingolo AD Inchingolo AM Use of dermal-fat grafts in the post-oncological reconstructive surgery of atrophies in the zygomatic region: clinical evaluations in the patients undergone to previous radiation therapy. Head Face Med. (2012) 8:33. 10.1186/1746-160X-8-33 23217096 PMC3527323

[30] Inchingolo F Tatullo M Abenavoli FM Marrelli M Inchingolo AD Inchingolo AM Comparison between traditional surgery, CO2 and nd:yag Laser treatment for generalized gingival hyperplasia in sturge-weber syndrome: a retrospective study. J Investig Clin Dent. (2010) 1:85–9. 10.1111/j.2041-1626.2010.00020.x 25427262

[31] Inchingolo AD Ceci S Patano A Inchingolo AM Montenegro V Di Pede C Elastodontic therapy of hyperdivergent class II patients using AMCOP® devices: a retrospective study. Appl Sci. (2022) 12:3259. 10.3390/app12073259

[32] McNamara JA. Maxillary transverse deficiency. Am J Orthod Dentofacial Orthop. (2000) 117:567–70. 10.1016/s0889-5406(00)70202-2 10799117

[33] Inchingolo F Santacroce L Cantore S Ballini A Del Prete R Topi S Probiotics and EpiCor® in human health. J Biol Regul Homeost Agents. (2019) 33:1973–9. 10.23812/19-543-L 31858774

[34] Patano A Cirulli N Beretta M Plantamura P Inchingolo AD Inchingolo AM Education technology in orthodontics and paediatric dentistry during the COVID-19 pandemic: a systematic review. Int J Environ Res Public Health. (2021) 18:6056. 10.3390/ijerph18116056 34199882 PMC8200064

[35] Cozza P Baccetti T Franchi L Mucedero M Polimeni A. Sucking habits and facial hyperdivergency as risk factors for anterior open bite in the mixed dentition. Am J Orthod Dentofacial Orthop. (2005) 128:517–9. 10.1016/j.ajodo.2005.04.032 16214636

[36] Warren JJ Slayton RL Bishara SE Levy SM Yonezu T Kanellis MJ. Effects of nonnutritive sucking habits on occlusal characteristics in the mixed dentition. Pediatr Dent. (2005) 27:445–50. 16532883

[37] Ciavarella D Tepedino M Laurenziello M Guida L Troiano G Montaruli G Swallowing and temporomandibular disorders in adults. J Craniofac Surg. (2018) 29:e262–7. 10.1097/SCS.0000000000004376 29554061

[38] Inchingolo AD Inchingolo AM Campanelli M Carpentiere V de Ruvo E Ferrante L Orthodontic treatment in patients with atypical swallowing and malocclusion: a systematic review. J Clin Pediatr Dent. (2024) 48:14–26. 10.22514/jocpd.2024.100 39275817

[39] Hanson M. A review of: variation of swallowing patterns with malocclusions, by Ibrahim A. Nashashibi (1987). Int J Orofac Myol. (1988) 14(2):17. 10.52010/ijom.1988.14.2.6 3481823

[40] Ngan P Fields HW. Open bite: a review of etiology and management. Pediatr Dent. (1997) 19:91–8. 9106869

[41] Graber LW Vanarsdall RL, Jr. Vig KWL Huang GJ. Orthodontics: current principles and techniques. J Indian Orthod Soc. (2017) 51(2):141. 10.4103/0301-5742.204613

[42] Lentini-Oliveira DA Carvalho FR Rodrigues CG Ye Q Prado LBF Prado GF Orthodontic and orthopaedic treatment for anterior open bite in children. Cochrane Database Syst Rev. (2014) 2014:CD005515. 10.1002/14651858.CD005515.pub3 25247473 PMC10964129

[43] Bonnet B. Un appareil de reposturation : l’Enveloppe Linguale nocturne (E.L.N.). Rev Orthop Dento Faciale. (1992) 26:329–47. 10.1051/odf/1992025

[44] Seehra J Fleming PS Mandall N DiBiase AT. A comparison of two different techniques for early correction of class III malocclusion. Angle Orthod. (2012) 82:96–101. 10.2319/032011-197.1 21806467 PMC8881034

[45] Giuntini V McNamara JA Franchi L. Treatment of class II malocclusion in the growing patient: early or late? Semin Orthod. (2023) 29:183–8. 10.1053/j.sodo.2023.04.008

[46] Artese F Fernandes LQP de Oliveira Caetano SR Miguel JAM. Early treatment for anterior open bite: choosing adequate treatment approaches. Semin Orthod. (2023) 29:207–15. 10.1053/j.sodo.2023.06.001

[47] Chandra S Jha AK. Early management of class III malocclusion in mixed dentition. Int J Clin Pediatr Dent. (2021) 14:331–4. 10.5005/jp-journals-10005-1752 34413617 PMC8343682

[48] Zhou C Duan P He H Song J Hu M Liu Y Expert consensus on pediatric orthodontic therapies of malocclusions in children. Int J Oral Sci. (2024) 16:32. 10.1038/s41368-024-00299-8 38627388 PMC11021504

[49] Baneshi M O’malley L El-angbawi A Thiruvenkatachari B. Effectiveness of clear orthodontic aligners in correcting malocclusions: a systematic review and meta-analysis. J Evid Based Dent Pract. (2025) 25:102081. 10.1016/j.jebdp.2024.102081 39947778

[50] Krishnaswamy NR. Vertical control with TADs: procedures and protocols. Semin Orthod. (2018) 24:108–22. 10.1053/j.sodo.2018.01.010

[51] Ghafari JG Haddad RV. Open bite: spectrum of treatment potentials and limitations. Semin Orthod. (2013) 19:239–52. 10.1053/j.sodo.2013.07.007

[52] Serafin M Fastuca R Caprioglio A. CBCT Analysis of dento-skeletal changes after rapid versus slow maxillary expansion on deciduous teeth: a randomized clinical trial. J Clin Med. (2022) 11:4887. 10.3390/jcm11164887 36013125 PMC9409744

[53] Lanteri V Abate A Cavagnetto D Ugolini A Gaffuri F Gianolio A Cephalometric changes following maxillary expansion with Ni-Ti leaf springs palatal expander and rapid maxillary expander: a retrospective study. Appl Sci. (2021) 11:5748. 10.3390/app11125748

[54] Kuroda S Sugawara Y Deguchi T Kyung H-M Takano-Yamamoto T. Clinical use of miniscrew implants as orthodontic anchorage: success rates and postoperative discomfort. Am J Orthod Dentofacial Orthop. (2007) 131:9–15. 10.1016/j.ajodo.2005.02.032 17208101

[55] Mummolo S Marchetti E Albani F Campanella V Pugliese F Di Martino S Comparison between rapid and slow palatal expansion: evaluation of selected periodontal indices. Head Face Med. (2014) 10:30. 10.1186/1746-160X-10-30 25128278 PMC4158002

[56] Lanteri V Cossellu G Gianolio A Beretta M Lanteri C Cherchi C Comparison between RME, SME and leaf expander in growing patients: a retrospective postero-anterior cephalometric study. Eur J Paediatr Dent. (2018) 19:199–204. 10.23804/ejpd.2018.19.03.6 30063151

[57] Nieri M Paoloni V Lione R Barone V Marino Merlo M Giuntini V Comparison between two screws for maxillary expansion: a multicenter randomized controlled trial on patient’s reported outcome measures. Eur J Orthod. (2021) 43:293–300. 10.1093/ejo/cjaa063 33215652

[58] Yılmaz A Arman-Özçırpıcı A Erken S Polat-Özsoy Ö. Comparison of short-term effects of Mini-implant-supported maxillary expansion appliance with two conventional expansion protocols. Eur J Orthod. (2015) 37:556–64. 10.1093/ejo/cju094 25564504

[59] Paoloni V Giuntini V Lione R Nieri M Barone V Merlo MM Comparison of the dento-skeletal effects produced by leaf expander versus rapid maxillary expander in prepubertal patients: a two-center randomized controlled trial. Eur J Orthod. (2022) 44:163–9. 10.1093/ejo/cjab035 34114608

[60] Ortu E Di Nicolantonio S Cova S Pietropaoli D De Simone L Monaco A. Efficacy of elastodontic devices in temporomandibular disorder reduction assessed by computer aid evaluation. Appl Sci. (2024) 14:1651. 10.3390/app14041651

[61] Dipalma G Inchingolo AD Cardarelli F Di Lorenzo A Viapiano F Ferrante L Effects of AMCOP® Elastodontic Devices on Skeletal Divergence and Airway Dimensions in Growing Patients. Available online at: https://www.mdpi.com/2077-0383/14/15/5297 (Accessed November 5, 2025). 10.3390/jcm14155297 PMC12347557 40806919

[62] Inchingolo AD Patano A Coloccia G Ceci S Inchingolo AM Marinelli G The efficacy of a new AMCOP® elastodontic protocol for orthodontic interceptive treatment: a case series and literature overview. Int J Environ Res Public Health. (2022) 19:988. 10.3390/ijerph19020988 35055811 PMC8775806

[63] Pennacchio BFP Giorgio RV Cardarelli F Sguera N Vecchio MD Memè L AMCOP Bio-Activators: an innovative solution in interceptive orthodontics for the treatment of malocclusions and orofacial dysfunctions. Oral Implantol (Rome). (2024) 16:162–75. 10.11138/oi163.1suppl162-175

[64] Manfredini D Ahlberg J Lobbezoo F. Bruxism definition: past, present, and future – what should a prosthodontist know? J Prosthet Dent. (2022) 28(5):905–12. 10.1016/j.prosdent.2021.01.026 33678438

[65] Kaur S Soni S Prashar A Bansal N Brar J Kaur M. Functional appliances. Indian J Dent Sci. (2017) 9:276. 10.4103/IJDS.IJDS_65_16

[66] Inchingolo AM Inchingolo AD Trilli I Ferrante L Di Noia A de Ruvo E Orthopedic devices for skeletal class III malocclusion treatment in growing patients: a comparative effectiveness systematic review. J Clin Med. (2024) 13:7141. 10.3390/jcm13237141 39685600 PMC11642149

[67] Gawali N Shah PP Gowdar IM Bhavsar KA Giri D Laddha R. The evolution of digital dentistry: a comprehensive review. J Pharm Bioallied Sci. (2024) 16:S1920–1922. 10.4103/jpbs.jpbs_11_24 39346228 PMC11426768

[68] Panayi NC Efstathiou S Christopoulou I Kotantoula G Tsolakis IA. Digital orthodontics: present and future. AJO-DO Clin Companion. (2024) 4:14–25. 10.1016/j.xaor.2023.12.001

[69] Davidovitch Z Krishnan V. Role of basic biological sciences in clinical orthodontics: a case series. Am J Orthod Dentofacial Orthop. (2009) 135:222–31. 10.1016/j.ajodo.2007.03.028 19201330

[70] Reitan K. Tissue behavior during orthodontic tooth movement. Am J Orthod. (1960) 46:881–900. 10.1016/0002-9416(60)90091-9

[71] Huettner RJ Young RW. The movability of vital and devitalized teeth in the macacus rhesus monkey. Am J Orthod. (1955) 41:594–603. 10.1016/0002-9416(55)90211-6 13236306

[72] Tomášik J Zsoldos M Oravcová Ľ Lifková M Pavleová G Strunga M AI And face-driven orthodontics: a scoping review of digital advances in diagnosis and treatment planning. AI. (2024) 5:158–76. 10.3390/ai5010009

[73] Ramdan K. Digital orthodontics: an overview. MSA Dent J. (2023) 2:26–30. 10.21608/msadj.2023.211756.1020

[74] Li Q Li S Fu D Liao G Zhou X Gong T The role of emerging digital technologies in revolutionizing dental education: a bibliometric analysis. J Dent Educ. (2025) 60(29):16044–50. 10.1002/jdd.70033 40899003

[75] Inchingolo F Patano A Inchingolo AM Riccaldo L Morolla R Netti A Analysis of mandibular muscle variations following condylar fractures: a systematic review. J Clin Med. (2023) 12(18):5925. 10.3390/jcm12185925 37762866 PMC10532393

[76] Nulton TJ Olex AL Dozmorov M Morgan IM Windle B. Analysis of the cancer genome atlas sequencing data reveals novel properties of the human papillomavirus 16 genome in head and neck squamous cell carcinoma. Oncotarget. (2017) 8:17684–99. 10.18632/oncotarget.15179 28187443 PMC5392278

[77] Arrigoni R Ballini A Santacroce L Cantore S Inchingolo A Inchingolo F Another Look at dietary polyphenols: challenges in cancer prevention and treatment. Curr Med Chem. (2022) 29:1061–82. 10.2174/0929867328666210810154732 34375181

[78] Sebastian P Babu JM Prathibha R Hariharan R Pillai MR. Anterior tongue cancer with No history of tobacco and alcohol use may be a distinct molecular and clinical entity. J Oral Pathol Med. (2014) 43:593–9. 10.1111/jop.12175 24809775

[79] Makarewicz J Kaźmierczak-Siedlecka K Sobocki BK Dobrucki IT Kalinowski L Stachowska E. Anti-cancer management of head and neck cancers and oral microbiome—what can we clinically obtain? Front Cell Infect Microbiol. (2024) 14:1329057. 10.3389/fcimb.2024.1329057 38481661 PMC10933093

[80] De Gabriele O Dallatana G Riva R Vasudavan S Wilmes B. The easy driver for placement of palatal Mini-implants and a maxillary expander in a single appointment. J Clin Orthod. (2017) 51:728–37. 29360638

[81] Inchingolo F Inchingolo AM Latini G Palmieri G Di Pede C Trilli I Application of graphene oxide in oral surgery: a systematic review. Materials. (2023) 16:6293. 10.3390/ma16186293 37763569 PMC10532659

[82] Dipalma G Inchingolo AD Inchingolo AM Piras F Carpentiere V Garofoli G Artificial intelligence and its clinical applications in orthodontics: a systematic review. Diagnostics. (2023) 13:3677. 10.3390/diagnostics13243677 38132261 PMC10743240

[83] Bartemes KR Gochanour BR Routman DM Ma DJ Doering KA Burger KN Assessing the capacity of methylated DNA markers of cervical squamous cell carcinoma to discriminate oropharyngeal squamous cell carcinoma in human papillomavirus mediated disease. Oral Oncol. (2023) 146:106568. 10.1016/j.oraloncology.2023.106568 37717549 PMC10591712

[84] Inchingolo F Inchingolo AM Latini G Ferrante L Trilli I Del Vecchio G Oxidative stress and natural products in orthodontic treatment: a systematic review. Nutrients. (2024) 16:113. 10.3390/nu16010113 PMC10780648 38201943

[85] Inchingolo F Inchingolo AM Fatone MC Avantario P Del Vecchio G Pezzolla C Management of rheumatoid arthritis in primary care: a scoping review. Int J Environ Res Public Health. (2024) 21:662. 10.3390/ijerph21060662 38928909 PMC11203333

[86] Cruz RS Lemos CAA de Luna Gomes JM Fernandes e Oliveira HF Pellizzer EP Verri FR. Clinical comparison between crestal and subcrestal dental implants: a systematic review and meta-analysis. J Prosthet Dent. (2022) 127:408–17. 10.1016/j.prosdent.2020.11.003 33358610

[87] Heintze SD Rousson V Hickel R. Clinical effectiveness of direct anterior restorations—a meta-analysis. Dent Mater. (2015) 31:481–95. 10.1016/j.dental.2015.01.015 25773188

[88] Pilloni A Rojas MA Trezza C Carere M De Filippis A Marsala RL Clinical effects of the adjunctive use of a polynucleotide and hyaluronic acid-based gel in the subgingival re-instrumentation of residual periodontal pockets: a randomized, split-mouth clinical trial. J Periodontol. (2023) 94(3):354–63. 10.1002/JPER.22-0225 36189651

[89] Pilloni A Rojas MA Trezza C Carere M De Filippis A Marsala RL Clinical Effects of the Adjunctive Use of Polynucleotide and Hyaluronic Acid-based Gel in the Subgingival Re-instrumentation of Residual Periodontal Pockets: A Randomized, Split-mouth Clinical Trial—Pilloni—2023—Journal of Periodontology—Wiley Online Library. Available online at: https://aap.onlinelibrary.wiley.com/doi/10.1002/JPER.22-0225 (Accessed March 9, 2025). 10.1002/JPER.22-0225 36189651

[90] Tadakamadla SK Bharathwaj VV Duraiswamy P Sforza C Tartaglia GM. Clinical efficacy of a new cetylpyridinium chloride-hyaluronic acid–based mouthrinse compared to chlorhexidine and placebo mouthrinses—a 21-day randomized clinical trial. Int J Dent Hyg. (2020) 18:116–23. 10.1111/idh.12413 31276312

[91] Das M Das AC Panda S Greco Lucchina A Mohanty R Manfredi B Clinical efficacy of grape seed extract as an adjuvant to scaling and root planing in treatment of periodontal pockets. J Biol Regul Homeost Agents. (2021) 35:89–96. 10.23812/21-2supp1-8 34281305

[92] Sandalli N Cildir S Guler N. Clinical investigation of traumatic injuries in yeditepe university, Turkey during the last 3 years. Dent Traumatol. (2005) 21:188–94. 10.1111/j.1600-9657.2005.00309.x 16026523

[93] Pilloni A Marini L Gagliano N Canciani E Dellavia C Cornaghi LB Clinical, histological, immunohistochemical, and biomolecular analysis of hyaluronic acid in early wound healing of human gingival tissues: a randomized, split-mouth trial. J Periodontol. (2023) 94:868–81. 10.1002/JPER.22-0338 36648006

[94] Paolantonio M D’Ercole S Pilloni A D’Archivio D Lisanti L Graziani F Clinical, microbiologic, and biochemical effects of subgingival administration of a Xanthan-based chlorhexidine gel in the treatment of periodontitis: a randomized multicenter trial. J Periodontol. (2009) 80:1479–92. 10.1902/jop.2009.090050 19722799

[95] Garbarino S Lanteri P Durando P Magnavita N Sannita WG. Co-morbidity, mortality, quality of life and the healthcare/welfare/social costs of disordered sleep: a rapid review. Int J Environ Res Public Health. (2016) 13:831. 10.3390/ijerph13080831 27548196 PMC4997517

[96] Gunjal S Pateel DGS. Comparative effectiveness of propolis with chlorhexidine mouthwash on gingivitis—a randomized controlled clinical study. BMC Complement Med Ther. (2024) 24:154. 10.1186/s12906-024-04456-8 38582863 PMC10998313

[97] Bolcato V Franzetti C Fassina G Basile G Martinez RM Tronconi LP. Comparative study on informed consent regulation in health care among Italy, France, United Kingdom, nordic countries, Germany, and Spain. J Forensic Leg Med. (2024) 103:102674. 10.1016/j.jflm.2024.102674 38502996

[98] Aparna K Naik S Naik MT Goud Padala R Malshetwar SS Kothamasu V. Vertical dysplasia. Int Dent J Stud Res. (2024) 12:110–6. 10.18231/j.idjsr.2024.022

[99] El Guennouni B Houb-Dine A Ben Mohimd H Zaoui F. Orthodontic treatment of deep bite in mixed dentition and/or early permanent dentition: what about stability?—a systematic review. Int Orthod. (2025) 23:100956. 10.1016/j.ortho.2024.100956 39742828

[100] 4. Management of Vertical Discrepancies (2) 2 | PDF | Orthodontics | Tooth. Available online at: https://it.scribd.com/presentation/415147508/4-Management-of-Vertical-Discrepancies-2-2 (Accessed November 5, 2025).

[101] Keski-Nisula K Hernesniemi R Heiskanen M Keski-Nisula L Varrela J. Orthodontic intervention in the early mixed dentition: a prospective, controlled study on the effects of the eruption guidance appliance. Am J Orthod Dentofacial Orthop. (2008) 133:254–60; quiz 328.e2. 10.1016/j.ajodo.2006.05.039 18249292

[102] Rozzi M Alesi G Mucedero M Cozza P. Dentoskeletal effects of rapid maxillary expander therapy in early mixed dentition patients with different vertical growing patterns without posterior crossbite: a retrospective study. Am J Orthod Dentofacial Orthop. (2023) 163:319–27. 10.1016/j.ajodo.2021.11.018 36443147

[103] Dipalma G Inchingolo AD Cardarelli F Di Lorenzo A Viapiano F Ferrante L Effects of AMCOP® elastodontic devices on skeletal divergence and airway dimensions in growing patients. J Clin Med. (2025) 14:5297. 10.3390/jcm14155297 40806919 PMC12347557

[104] Shapiro PA. Stability of open bite treatment. Am J Orthod Dentofacial Orthop. (2002) 121:566–8. 10.1067/mod.2002.124175 12080301

[105] Kim YH. Anterior openbite and its treatment with multiloop edgewise archwire. Angle Orthod. (1987) 57:290–321. 10.1043/0003-3219(1987)057/3C0290:AOAITW/3E2.0.CO;2 3479033

[106] Nasry HA Barclay SC. Periodontal lesions associated with deep traumatic overbite. Br Dent J. (2006) 200:557–61; quiz 588. 10.1038/sj.bdj.4813587 16732243

[107] Proffit WR. The timing of early treatment: an overview. Am J Orthod Dentofacial Orthop. (2006) 129:S47–49. 10.1016/j.ajodo.2005.09.014 16644417

[108] Harris EF Butler ML. Patterns of incisor root resorption before and after orthodontic correction in cases with anterior open bites. Am J Orthod Dentofacial Orthop. (1992) 101:112–9. 10.1016/0889-5406(92)70002-R 1739065

[109] Kaje R Rashme R Manimegalan P Vundela RR Saidalavi SK Jadhav AV. Assessing the efficacy of early versus late orthodontic intervention in the management of class II malocclusion: a comparative analysis. J Pharm Bioallied Sci. (2024) 16:S2691–3. 10.4103/jpbs.jpbs_370_24 39346279 PMC11426744

[110] Valério P Poklepović Peričić T Rossi A Grippau C Tavares Campos JDS Borges Do Nascimento IJ. The effectiveness of early intervention on malocclusion and its impact on craniofacial growth: a systematic review. Contemp Pediatr Dent. (2021) 2:1–18. 10.51463/cpd.2021.61

[111] Mangano F. Digital dentistry. J Dent. (2021) 109:103693. 10.1016/j.jdent.2021.103693 34004272

[112] Genovese P Giambò P Abramo F Mancini M Pastore M D’Amico C. Advancements and applications in digital dentistry: a scoping review. In: Badnjević A Gurbeta Pokvić L, editors. Proceedings of the MEDICON’23 and CMBEBIH’23. Cham: Springer Nature Switzerland (2024). p. 702–9.

[113] Hellman M. Open-Bite. Int J Orthodontia Oral Surg Radiogr. (1931) 17:421–44. 10.1016/S0099-6963(31)80143-7

[114] Matsumoto MAN Romano FL Ferreira JTL Valério RA. Open bite: diagnosis, treatment and stability. Braz Dent J. (2012) 23:768–78. 10.1590/S0103-64402012000600024 23338275

[115] Xun C Zeng X Wang X. Microscrew anchorage in skeletal anterior open-bite treatment. Angle Orthod. (2007) 77:47–56. 10.2319/010906-14R.1 17029531

[116] Sugawara J Baik UB Umemori M Takahashi I Nagasaka H Kawamura H Treatment and posttreatment dentoalveolar changes following intrusion of mandibular molars with application of a skeletal anchorage system (SAS) for open bite correction. Int J Adult Orthodon Orthognath Surg. (2002) 17:243–53. 12592995

[117] Jedliński M Mazur M Grocholewicz K Janiszewska-Olszowska J. 3D Scanners in orthodontics—current knowledge and future perspectives—a systematic review. Int J Environ Res Public Health. (2021) 18:1121. 10.3390/ijerph18031121 33513981 PMC7908072

[118] Rossini G Parrini S Castroflorio T Deregibus A Debernardi CL. Efficacy of clear aligners in controlling orthodontic tooth movement: a systematic review. Angle Orthod. (2015) 85(5):881–9. 10.2319/061614-436.1 25412265 PMC8610387

[119] Bondemark L. A comparative analysis of distal maxillary molar movement produced by a new lingual intra-arch Ni-Ti coil appliance and a magnetic appliance. Eur J Orthod. (2000) 22:683–95. 10.1093/ejo/22.6.683 11212604

[120] Ke Y Zhu Y Zhu M. A comparison of treatment effectiveness between clear aligner and fixed appliance therapies. BMC Oral Health. (2019) 19(1):24. 10.1186/s12903-018-0695-z 30674307 PMC6343314

[121] Ferguson DJ Carano A Bowman SJ Davis EC Gutierrez Vega ME Lee SH. A comparison of two maxillary molar distalizing appliances with the distal jet. World J Orthod. (2005) 6:382–90. 16379210

[122] Yazdi M Daryanavard H Ashtiani AH Moradinejad M Rakhshan V. A systematic review of biocompatibility and safety of orthodontic clear aligners and transparent vacuum-formed thermoplastic retainers: bisphenol-A release, adverse effects, cytotoxicity, and estrogenic effects. Dent Res J (Isfahan). (2023) 20:41. 10.4103/1735-3327.372658 37180685 PMC10166753

[123] Keser E Naini FB. Accelerated orthodontic tooth movement: surgical techniques and the regional acceleratory phenomenon. Maxillofac Plast Reconstr Surg. (2022) 44:1. 10.1186/s40902-021-00331-5 34984554 PMC8727645

[124] Lyu X Cao X Chen L Liu Y Li H Hu C Accumulated biomechanical effects of mandibular molar mesialization using clear aligners with auxiliary devices: an iterative finite element analysis. Prog Orthod. (2023) 24:13. 10.1186/s40510-023-00462-7 37032410 PMC10083150

[125] Pasciuti E, Coloccia G, Inchingolo AD, Patano A, Ceci S, Bordea IR, et al. Applied Sciences | Free Full-Text | Deep Bite Treatment with Aligners: A New Protocol. Available online at: https://www.mdpi.com/2076-3417/12/13/6709 (Accessed October 5, 2023).

[126] Inchingolo AD Ceci S Patano A Inchingolo AM Montenegro V Pede CD Applied Sciences | Free Full-Text | Elastodontic Therapy of Hyperdivergent Class II Patients Using AMCOP® Devices: A Retrospective Study. Available online at: https://www.mdpi.com/2076-3417/12/7/3259 (Accessed November 2, 2023).

[127] Malcangi G Inchingolo AD Patano A Coloccia G Ceci S Garibaldi M Applied Sciences | Free Full-Text | Impacted Central Incisors in the Upper Jaw in an Adolescent Patient: Orthodontic-Surgical Treatment—A Case Report. Available online at: https://www.mdpi.com/2076-3417/12/5/2657 (Accessed October 1, 2023).

[128] Kinzinger GSM Gross U Fritz UB Diedrich PR. Anchorage quality of deciduous molars versus premolars for molar distalization with a Pendulum appliance. Am J Orthod Dentofacial Orthop. (2005) 127:314–23. 10.1016/j.ajodo.2004.09.014 15775946

[129] Loberto S Paoloni V Pavoni C Cozza P Lione R. Anchorage loss evaluation during maxillary molars distalization performed by clear aligners: a retrospective study on 3D digital casts. Appl Sci. (2023) 13:3646. 10.3390/app13063646

[130] Urdiales-Gálvez F Martín-Sánchez S Maíz-Jiménez M Castellano-Miralla A Lionetti-Leone L. Concomitant use of hyaluronic acid and Laser in facial rejuvenation. Aesthetic Plast Surg. (2019) 43:1061–70. 10.1007/s00266-019-01393-7 31073742 PMC6742610

[131] Vainio L. Connection between movements of mouth and hand: perspectives on development and evolution of speech. Neurosci Biobehav Rev. (2019) 100:211–23. 10.1016/j.neubiorev.2019.03.005 30871957

[132] Liew S Wu WTL Chan HH Ho WWS Kim H-J Goodman GJ Consensus on changing trends, attitudes, and concepts of Asian beauty. Aesthetic Plast Surg. (2016) 40:193–201. 10.1007/s00266-015-0562-0 26408389 PMC4819477

[133] Raggio BS Brody-Camp SA Jawad BA Winters RD Aslam R. Complications associated with medical tourism for facial rejuvenation: a systematic review. Aesthetic Plast Surg. (2020) 44:1058–65. 10.1007/s00266-020-01638-w 32040602

[134] Choi S-H Shi K-K Cha J-Y Park Y-C Lee K-J. Nonsurgical miniscrew-assisted rapid maxillary expansion results in acceptable stability in young adults. Angle Orthod. (2016) 86:713–20. 10.2319/101415-689.1 26938955 PMC8600851

[135] Malcangi G Patano A Palmieri G Riccaldo L Pezzolla C Mancini A Oral piercing: a pretty risk—a scoping review of local and systemic complications of this current widespread fashion. Int J Environ Res Public Health. (2023) 20:5744. 10.3390/ijerph20095744 37174261 PMC10177791

[136] Dinoi MT Marchetti E Garagiola U Caruso S Mummolo S Marzo G. Orthodontic treatment of an unerupted mandibular canine tooth in a patient with mixed dentition: a case report. J Med Case Rep. (2016) 10:170. 10.1186/s13256-016-0923-6 27286815 PMC4902959

[137] Gibson BC Claus ED Sanguinetti J Witkiewitz K Clark VP. A review of functional brain differences predicting relapse in substance use disorder: actionable targets for new methods of noninvasive brain stimulation. Neurosci Biobehav Rev. (2022) 141:104821. 10.1016/j.neubiorev.2022.104821 35970417

[138] Bell RA. A review of maxillary expansion in relation to rate of expansion and patient’s age. Am J Orthod. (1982) 81:32–7. 10.1016/0002-9416(82)90285-8 6758589

[139] Lo Russo L Caradonna G Salamini A Guida L. A single procedure for the registration of maxillo-mandibular relationships and alignment of intraoral scans of edentulous maxillary and mandibular arches. J Prosthodont Res. (2020) 64:55–9. 10.1016/j.jpor.2019.04.009 31101518

[140] Hadavi F Caffesse RG Charbeneau GT. A study of the gingival response to polished and unpolished amalgam restorations. J Can Dent Assoc. (1986) 52:211–4. 3513918

[141] Adam FA Mohd N Rani H Mohd Yusof MYP Baharin B. A systematic review and meta-analysis on the comparative effectiveness of Salvadora Persica—extract mouthwash with chlorhexidine gluconate in periodontal health. J Ethnopharmacol. (2023) 302:115863. 10.1016/j.jep.2022.115863 36283639

[142] Ogordi P Ize-Iyamu I. A ten year audit of traumatic dental injuries in children in a tertiary hospital in southern Nigeria. Niger J Dent Res. (2020) 5:177.

[143] Zhang J-L Peng Z-L Huang J Pan Y-J Sun Z-W Mai Z-H. A two-year retrospective study on traumatic dental injury in the primary dentition. Medicine (Baltimore). (2023) 102:e35750. 10.1097/MD.0000000000035750 37960738 PMC10637491

[144] West TE Ernst RK Jansson-Hutson MJ Skerrett SJ. Activation of toll-like receptors by Burkholderia Pseudomallei. BMC Immunol. (2008) 9:46. 10.1186/1471-2172-9-46 18691413 PMC2527550

[145] Boeckel DG Sesterheim P Peres TR Augustin AH Wartchow KM Machado DC Adipogenic mesenchymal stem cells and hyaluronic acid as a cellular compound for bone tissue engineering. J Craniofac Surg. (2019) 30:777–83. 10.1097/SCS.0000000000005392 30865107

[146] Avila ER Williams SE Disselhorst-Klug C. Advances in EMG measurement techniques, analysis procedures, and the impact of muscle mechanics on future requirements for the methodology. J Biomech. (2023) 156:111687. 10.1016/j.jbiomech.2023.111687 37339541

[147] Kim S-H Kim KB. Advances in maxillary transverse deficiency treatment. Semin Orthod. (2025) 31:177–8. 10.1053/j.sodo.2024.12.004

[148] Inchingolo AD Patano A Coloccia G Ceci S Inchingolo AM Marinelli G Treatment of class III malocclusion and anterior crossbite with aligners: a case report. Medicina. (2022) 58:603. 10.3390/medicina58050603 35630020 PMC9147027

[149] Baccetti T Franchi L Cameron CG McNamara JA. Treatment timing for rapid maxillary expansion. Angle Orthod. (2001) 71:343–50. 10.1043/0003-3219(2001)071/3C0343:TTFRME/3E2.0.CO;2 11605867

[150] Peters MDJ Marnie C Tricco AC Pollock D Munn Z Alexander L Updated methodological guidance for the conduct of scoping reviews. JBI Evid Synth. (2020) 18:2119–26. 10.11124/JBIES-20-00167 33038124

[151] Mummolo S Nota A Marchetti E Padricelli G Marzo G. The 3D tele motion tracking for the orthodontic facial analysis. Biomed Res Int. (2016) 2016:4932136. 10.1155/2016/4932136 28044130 PMC5156870

[152] Merheb J Vercruyssen M Coucke W Beckers L Teughels W Quirynen M. The fate of buccal bone around dental implants. A 12-month postloading follow-up study. Clin Oral Implants Res. (2017) 28:103–8. 10.1111/clr.12767 26749417

[153] Lanteri C Beretta M Lanteri V Gianolio A Cherchi C Franchi L. The leaf expander for non-compliance treatment in the mixed dentition. J Clin Orthod. (2016) 50:552–60. 27809214

[154] Page MJ McKenzie JE Bossuyt PM Boutron I Hoffmann TC Mulrow CD The PRISMA 2020 statement: an updated guideline for reporting systematic reviews. Br Med J. (2021) 372:n71. 10.1136/bmj.n71 33782057 PMC8005924

[155] Quinzi V Ferro R Rizzo F Marranzini EM Federici Canova F Mummolo S The two by four appliance: a nationwide cross-sectional survey. Eur J Paediatr Dent. (2018) 19(2):145–50. 10.23804/ejpd.2018.19.02.09 29790779

[156] Cossellu G Ugolini A Beretta M Farronato M Gianolio A Maspero C Three-Dimensional evaluation of slow maxillary expansion with leaf expander vs. Rapid maxillary expansion in a sample of growing patients: direct effects on maxillary arch and spontaneous mandibular response. Appl Sci. (2020) 10:4512. 10.3390/app10134512

[157] Tausche E Hansen L Hietschold V Lagravére MO Harzer W. Three-Dimensional Evaluation of Surgically Assisted Implant Bone-Borne Rapid Maxillary Expansion: A Pilot Study—ScienceDirect. Available online at: https://www.sciencedirect.com/science/article/pii/S0889540606015071?via/3Dihub (Accessed June 27, 2023). 10.1016/j.ajodo.2006.07.021 17448393

[158] Khosravi M Ugolini A Miresmaeili A Mirzaei H Shahidi-Zandi V Soheilifar S Tooth-Borne versus Bone-Borne Rapid Maxillary Expansion for Transverse Maxillary Deficiency: A Systematic Review—PubMed. Available online at: https://pubmed.ncbi.nlm.nih.gov/31280998/ (Accessed June 26, 2023). 10.1016/j.ortho.2019.06.003 31280998

